# Tumor Imaging Using Radiolabeled Matrix Metalloproteinase–Activated Anthrax Proteins

**DOI:** 10.2967/jnumed.119.226423

**Published:** 2019-10

**Authors:** Mary-Ann Elvina Xavier, Shihui Liu, Thomas H. Bugge, Julia Baguña Torres, Michael Mosley, Samantha L. Hopkins, Phillip D. Allen, Georgina Berridge, Iolanda Vendrell, Roman Fischer, Veerle Kersemans, Sean Smart, Stephen H. Leppla, Bart Cornelissen

**Affiliations:** 1 Department of Oncology, CRUK/MRC Oxford Institute for Radiation Oncology, University of Oxford, Oxford, United Kingdom; 2 Proteases and Tissue Remodeling Section, National Institute of Dental and Craniofacial Research, National Institutes of Health, Bethesda, Maryland; 3 Microbial Pathogenesis Section, Laboratory of Parasitic Diseases, National Institute of Allergy and Infectious Diseases, National Institutes of Health, Bethesda, Maryland; and; 4 Target Discovery Institute, Nuffield Department of Medicine, University of Oxford, Oxford, United Kingdom

**Keywords:** MMP, SPECT, cancer, pretargeting, anthrax lethal toxin

## Abstract

Increased activity of matrix metalloproteinases (MMPs) is associated with worse prognosis in different cancer types. The wild-type protective antigen (PA-WT) of the binary anthrax lethal toxin was modified to form a pore in cell membranes only when cleaved by MMPs (to form PA-L1). Anthrax lethal factor (LF) is then able to translocate through these pores. Here, we used a ^111^In-radiolabeled form of LF with the PA/LF system for noninvasive in vivo imaging of MMP activity in tumor tissue by SPECT. **Methods:** MMP-mediated activation of PA-L1 was correlated to anthrax receptor expression and MMP activity in a panel of cancer cells (HT1080, MDA-MB-231, B8484, and MCF7). Uptake of ^111^In-radiolabeled PA-L1, ^111^In-PA-WT^K563C^, or ^111^In-LF^E687A^ (a catalytically inactive LF mutant) in tumor and normal tissues was measured using SPECT/CT imaging in vivo. **Results:** Activation of PA-L1 in vitro correlated with anthrax receptor expression and MMP activity (HT1080 > MDA-MB-231 > B8484 > MCF7). PA-L1–mediated delivery of ^111^In-LF^E687A^ was demonstrated and was corroborated using confocal microscopy with fluorescently labeled LF^E687A^. Uptake was blocked by the broad-spectrum MMP inhibitor GM6001. In vivo imaging showed selective accumulation of ^111^In-PA-L1 in MDA-MB-231 tumor xenografts (5.7 ± 0.9 percentage injected dose [%ID]/g) at 3 h after intravenous administration. ^111^In-LF^E687A^ was selectively delivered to MMP-positive MDA-MB-231 tumor tissue by MMP-activatable PA-L1 (5.98 ± 0.62 %ID/g) but not by furin-cleavable PA-WT (1.05 ± 0.21 %ID/g) or a noncleavable PA variant control, PA-U7 (2.74 ± 0.24 %ID/g). **Conclusion:** Taken together, our results indicate that radiolabeled forms of mutated anthrax lethal toxin hold promise for noninvasive imaging of MMP activity in tumor tissue.

Matrix metalloproteinases (MMPs) are a family of zinc‐dependent proteases that together can degrade most components of the extracellular matrix. MMP activity is essential for tumor growth, allowing cancer cells to invade surrounding tissues and metastasize, as well as interact with the local tumor immune environment (1). The roles of MMPs include proteolytic degradation of the extracellular matrix, modification of cell–cell and cell–extracellular matrix interactions, stromal interactions, tumor cell migration, epithelial-to-mesenchymal transition, and angiogenesis. The structure, regulation, and substrates of MMPs have been extensively reviewed elsewhere (2). In particular, the gelatinases MMP2 and MMP9, as well as the membrane-anchored MMP14, have been strongly implicated in these processes. As such, overexpression of these biomarkers has been proposed to correlate with a cancer’s overall aggressiveness (1). In addition to their role in cancer, MMPs are also implicated in cardiovascular diseases, neurodegenerative disorders such as Alzheimer disease or Parkinson disease, autoimmune diseases, and several pulmonary and oral pathologic conditions (3). Although early attempts to target MMPs using small-molecule inhibitors for cancer therapy were largely unsuccessful in clinical trials (4), MMPs remain a viable and highly desirable therapeutic target in view of promising preclinical results and their role in disease progression.

A molecular imaging agent that allows noninvasive imaging of MMP activity and related proteins would allow visualization of these disease processes in vivo, permitting early detection, prognosis, and measurement of therapy efficacy. A diverse range of molecular imaging agents has been developed over the past few years, expertly summarized in a previous publication (5). This range includes agents to be used with a wide variety of molecular imaging modalities (SPECT, PET, MRI, and optical imaging). The agents are based on a variety of targeting vector systems, including labeled small-molecule MMP inhibitors (6), antibodies targeting MMPs (7), or activatable, cleavable peptides (8). With the exception of the last of these, all of these imaging agents bind in a 1-to-1 fashion to mostly soluble MMP enzymes. In addition, regarding pharmacokinetics and blood clearance, MMP specificity, and bioavailability, these compounds seldom reach sufficient tumor uptake to be useful as MMP imaging probes (5). Notably, these imaging agents generally do not distinguish between the noncatalytically active preforms and the active forms of MMPs. In contrast, Jiang et al. (8) reported a different approach, using so-called activatable imaging agents, consisting of a fluorescently labeled cell-penetrating nona-arginine peptide, quenched by a negatively charged poly-glutamate chain, attached via an MMP-cleavable linker sequence. On cleavage of the linker by MMPs, the cell-penetrating peptide is taken up by the tissue.

Here, we utilized a modified anthrax lethal toxin (LT) system to visualize MMP activity in tumor tissue using an adapted pretargeting system (Fig. 1) (9). *Bacillus anthracis* is a spore-forming bacterium that causes anthrax. As a means of suppressing its host’s immune response, the bacterium produces a set of toxins to promote its own survival: protective antigen (PA), lethal factor (LF), and edema factor. After binding to the ubiquitous anthrax receptors (CMG2 and TEM8), full-length PA (83 kDa) is cleaved by furin or furinlike proteases to a 63-kDa isoform (9). Thus, the PA is activated to form a hepta- or octameric prepore, creating a de novo binding site for LF and edema factor on the interface between cleaved PA monomers. LF is then threaded through the oligo-PA pore and is delivered to the cytoplasm, where it cleaves the N-terminus from several MEKs, thereby preventing the activation of Erk1/2, p38, and Jnk pathways, whereby it exerts its cytotoxic effects.

**FIGURE 1.**
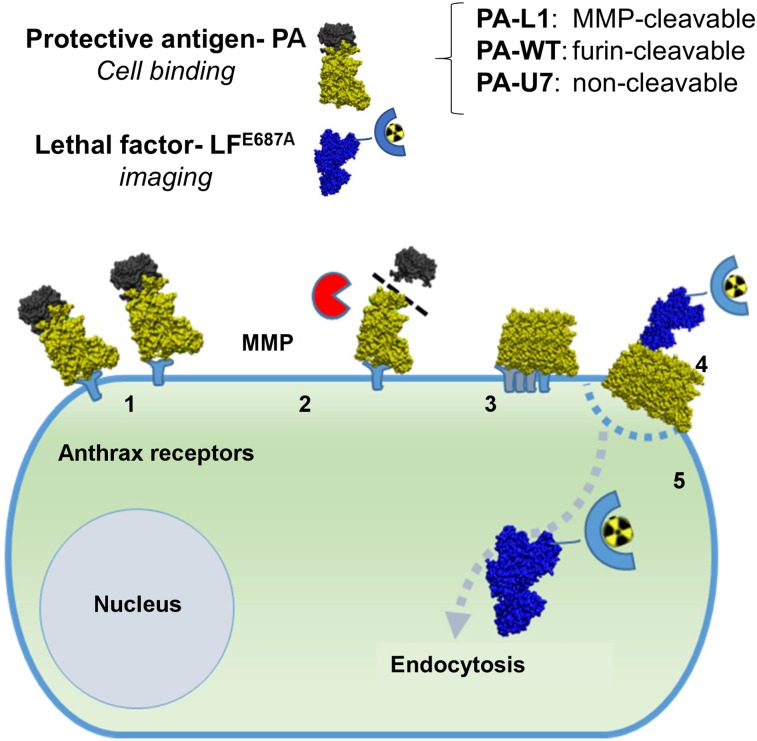
Schematic overview of MMP-activated pretargeting of cancer cells using PA-L1/LF system: binding of PA-L1 to anthrax receptors (1), cleavage and activation of PA-L1 by MMPs (2), formation of prepore (3), binding of ^111^In-LF^E687A^ to PA prepore and formation of PA pore (4), and endocytosis and delivery of ^111^In-LF^E687A^ to cytoplasm (5).

Liu et al. previously generated an engineered PA that requires cleavage-mediated activation by MMPs, by modifying the amino acid sequence that acts as a substrate for furin cleavage (10). An MMP-cleavable version, PA-L1, was generated by inserting an MMP-liable sequence, GPLGMLSQ, between amino acids S_168_–P_176_ of the original PA (wild-type PA, or PA-WT). PA-L1 allows MMP-selective intoxication of tumor cells in vitro, as well as tumor xenografts grown in mice, with a modified LF fusion toxin, incorporating the adenosine diphosphate ribosylation domain of *Pseudomonas* exotoxin A (FP59) (11).

Here, we expand the use of this MMP-activatable system to allow molecular imaging of MMP activity in mouse models of cancer, by SPECT imaging, using a radiolabeled catalytically inactive version of LF, LF^E687A^, in combination with MMP-activatable PA-L1. In this report, we show that this novel pretargeted imaging system is selective for MMP-expressing cancer cell lines in vitro, and we show selective, noninvasive, in vivo imaging in MMP-expressing tumor xenografts grown in mice.

## MATERIALS AND METHODS

### Protein Production and Synthesis of Labeled Compounds

All components of *B. anthracis* LT were expressed and purified as previously described (12). All protein batches were analyzed by liquid chromatography mass spectrometry analysis and sodium dodecyl sulfate polyacrylamide gel electrophoresis to confirm purity.

PA-WT (83 kDa) is cleavable by furin and other furinlike enzymes (13). Here, we used a PA-WT variant containing an engineered cysteine (PA-WT^K563C^; 83 kDa) whenever radiolabeled PA-WT is used (14). PA-L1 has the furin cleavage site replaced by a sequence targeted by MMPs (such as MMP2, MMP9, or MMP14) (PA-L1; 83 kDa) (10), whereas in PA-U7 an uncleavable sequence was inserted (PA-U7; 83 kDa) (15). LF variants included the fusion toxin of the N-terminal translocation domain of LF (LFn, LF amino acids 1–254) and *P. aeruginosa* exotoxin A domain III (FP59; 53 kDa) (15), LFn modified with a cysteine residue at the C terminus (LFn; 30 kDa) (16), and full-length mutant LF^E687A^ (90 kDa) containing a defective catalytic domain (17). Cleavage PA protein was confirmed by sodium dodecyl sulfate polyacrylamide gel electrophoresis after exposure to MMP2, furin. Full experimental details are laid out in the supplemental materials (available at http://jnm.snmjournals.org).

LFn and PA-WT^K563C^ were site-specifically conjugated to the metal ion chelator diethylenetriamine pentaacetic acid (DTPA), using the bifunctional agent maleimide-DTPA (2,2′-(1-carboxy-2-(carboxymethyl)-13-(2,5-dioxo-2,5-dihydro-1H-pyrrol-1-yl)-10-oxo-2,5,8,11-tetraazatridecane-5,8-diyl)diacetic acid) to allow radiolabeling with ^111^In, whereas PA-L1 or LF^E687A^ was conjugated to *p*-SCN-benzyl-DTPA (*S*-2-(4-isothiocyanatobenzyl)-DTPA) before radiolabeling with ^111^In, using previously described methods (18). For cross-validation of the radiolabeled compound using confocal fluorescence microscopy, LF^E687A^ was also fluorescently labeled using a Cy3-*N*-hydroxysuccinimide ester. Full experimental details are laid out in the supplemental materials.

### In Vitro Analyses

MCF-7, MDA-MB-231, and HT1080 cells were originally purchased from ATCC. KPC-mouse–derived B8484 cells were provided by Professor Owen Sansom at the CRUK Beatson Institute. Cells were cultured in Dulbecco modified Eagle medium, supplemented with 10% fetal bovine serum, 2 mM l-glutamine, 100 units/mL penicillin, and 0.1 mg/mL streptomycin, in a 37°C environment containing 5% CO_2_. Cells were authenticated by the provider, and the identities of purchased cells were corroborated by STR profiling. The cumulative length of culture was less than 3 mo after retrieval from liquid nitrogen storage. Cells were tested regularly to confirm the absence of *Mycoplasma* contamination.

Gelatin zymography analysis was used to gauge the level of MMP2 and MMP9 secretion of cells. MMP14 and CMG2 expression was corroborated by Western blot and immunofluorescence. Full experimental details are laid out in the supplemental materials.

The ability of the different PA variants to permit LF intoxication of the panel of cell lines was determined by MTT assay (3-(4,5-dimethylthiazol-2-yl)-2,5-diphenyltetrazolium bromide) after exposure of cells to PA-WT, PA-WT^K563C^, PA-L1, or PA-U7 in combination with FP59 (19). To investigate PA activation by MMP hydrolysis, cells were exposed to the broad-spectrum MMP inhibitor, GM6001 (30 μM; Abcam), for 30 min before adding PA/LF variants. Full experimental details are laid out in the supplemental materials.

Cy3-LF^E687A^ was added to HT1080 or MCF7 cells growing on glass cover slides, in combination with PA-WT, PA-L1, or a vehicle control. After 3 h, cells were washed, fixed, and mounted with 4′,6-diamidino-2-phenylindole, and images were acquired using a confocal microscope (TCS SP8; Leica). Full experimental details are laid out in the supplemental materials.

Saturation cell binding experiments were performed to determine the apparent dissociation constant (K_D_) and number of binding sites for ^111^In-PA-WT^K563C^ or ^111^In-PA-L1. In vitro assays were performed to evaluate the capacity of ^111^In-LF^E687A^ or ^111^In-LFn to interact with PA pores and to be delivered into cells after radiolabeling. Full experimental details are laid out in the supplemental materials.

### In Vivo Studies: Imaging and Biodistribution

All animal procedures were performed in accordance with the U.K. Animals (Scientific Procedures) Act of 1986 and with local ethical committee approval. Animals were housed in individually ventilated cages in sex-matched groups of up to 6 per cage in an artificial day–night cycle facility with ad libitum access to food and water. No animals were euthanized for welfare reasons. All analyses were performed masked to experimental group assignment.

To measure plasma clearance, dynamic SPECT/CT imaging of tumor-naïve SCID mice was performed after intravenous administration of ^111^In-LFn (3 μg, 3 MBq; 100 μL) or ^111^In-LF^E687E^ (10 μg, 10 MBq; 100 μL) through a cannula inserted in the tail vein. SPECT/CT images were acquired for up to 3 h in list mode using a VECTor^4^CT scanner (MILabs). Three mice were used per group. Additional experimental detail is provided in the supplemental materials.

To determine the pharmacokinetics of PA-L1 in vivo, dynamic SPECT images were acquired in MDA-MB-231 tumor–bearing mice over 3 h after intravenous injection of ^111^In-PA-L1 or ^111^In-PA-WT^K563C^ (20 μg, 10 MBq; 100 μL). Three mice were used per group.

MMP-activatable, PA-L1–mediated uptake of radiolabeled LF was evaluated in a pretargeting-inspired setup. MDA-MB-231 tumor xenograft–bearing mice were administered ^111^In-LF^E687E^ (10 μg, 10 MBq; 50 μL) by intravenous injection, together with PA-L1 (20 μg; 50 μL). To determine the PA-L1 selectivity of tumor uptake, and thus of MMP-mediated uptake, groups of control animals were administered ^111^In-LF^E687A^ alone or in combination with PA-WT (20 μg; 50 μL), PA-U7 (20 μg; 50 μL), or PA-L1 and an excess of unlabeled LF^E687A^ (1 mg; 100 μL). SPECT imaging was performed 24 h after administration of the ^111^In-LF/PA combinations. Three mice were used per group.

After imaging, the mice were euthanized by cervical dislocation, and selected organs, tissues, and blood were removed. The amount of ^111^In was determined per gram of tissue, normalized by the administered amount of ^111^In (expressed as percentage injected dose per gram [%ID/g]). Tumor tissues were stored in 30% sucrose and, after 24 h, flash-frozen with dry ice and stored at −80°C until required for further processing.

### Statistical Analysis

All statistical and regression analyses were performed using GraphPad Prism, version 7 (GraphPad Software). Linear regression was used to test for correlations between measurements. After testing for normality using a Shapiro–Wilk test, means were compared using a *t* test with the Welch correction for nonequal variances to compare 2 groups. One-way ANOVA followed by Dunnett posttests were used to compare multiple groups. Two-way ANOVA was used to analyze grouped data. All results are reported as mean ± SD of at least 3 independent replicates, unless otherwise indicated.

## RESULTS

### Cleavage and Activation of PA-L1 by MMP2 and MMP14

In a cell-free setting, recombinant human MMP2 and furin-activated MMP14, but not furin alone, were able to cleave PA-L1. Furin, but not MMP2, was able to cleave PA-WT (Supplemental Fig. 1). Cleavage of PA-L1 by MMP2 and MMP14 at the intended site was further confirmed by mass spectrometry (Supplemental Fig. 2).

### MMP-Mediated Delivery by PA-L1 of LF Variants into Cells

**FIGURE 2.**
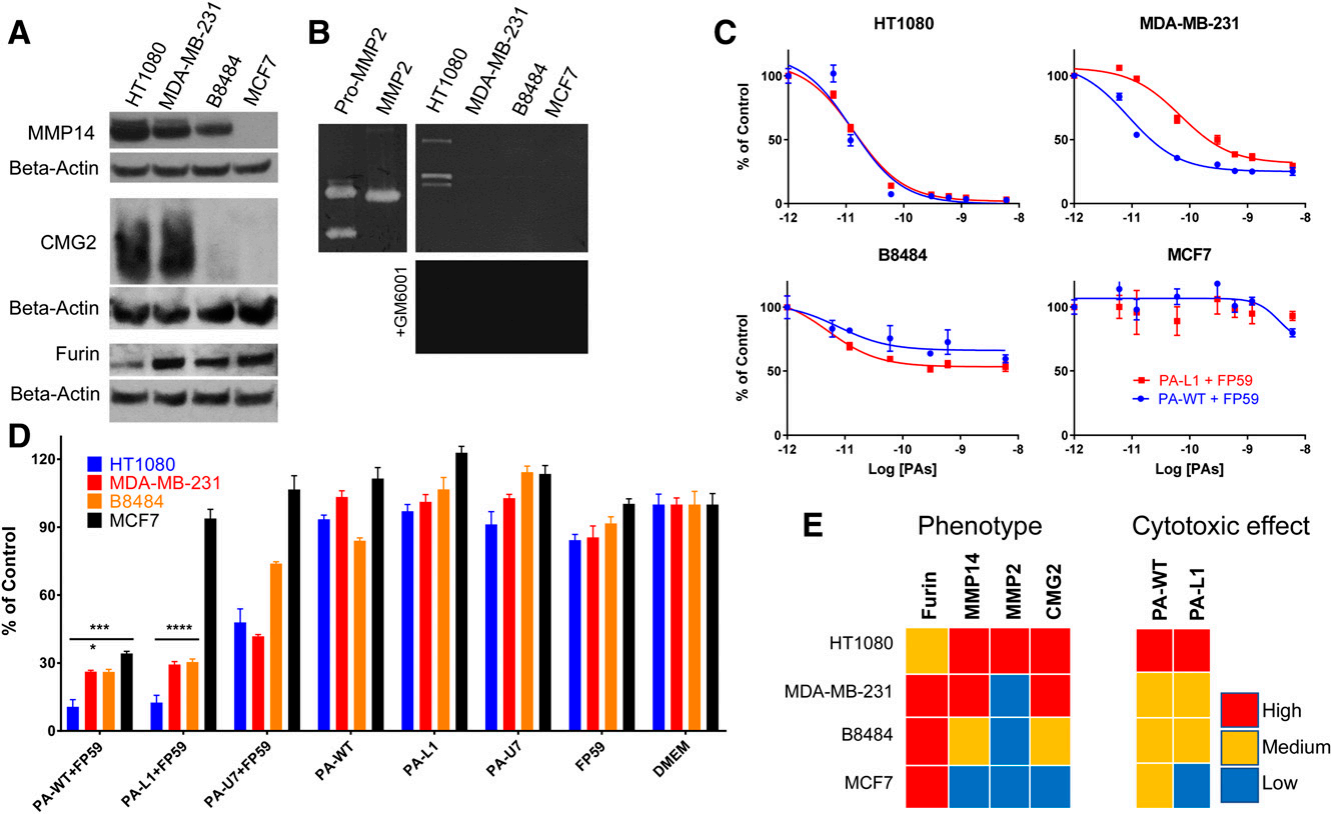
In vitro characterization of panel of cancer cell lines for protease and anthrax toxin receptor expression. (A) Western blots show differential MMP14, CMG2, and furin expression in whole-cell lysates. (B) MMP2 activity was measured in HT1080 cell lysates, using gelatin zymography. Broad-spectrum inhibitor GM6001 inhibited MMP2 activity. (C and D) Furin-cleavable PA-WT and MMP-cleavable PA-L1 delivered cytotoxic fusion toxin FP59 to panel of cancer cells, with variable efficiency. Controls are defined as MTT signal derived from vehicle-treated cells. *P < 0.05. ***P < 0.001.****P < 0.0001. (E) Comparison of results obtained in panels A–D.

A panel of cancer cell lines was characterized for the expression of various proteins involved in the *Bacillus anthracis* MMP-activatable LT system. All cell lines, except for HT1080, expressed relatively high levels of furin (Fig. 2A). Both HT1080 and MDA-MB-231 presented high levels of CMG2 and MMP14 expression. B8484 was found to have less CMG2 and MMP14 expression, whereas levels in MCF7 cells were undetectable by this technique. These findings were corroborated by immunohistochemistry in MDA-MB-231 and MCF7 cells (Supplemental Fig. 3). Gelatin zymography probing for MMP2 and MMP9 activity showed high MMP2 levels in HT1080, with far lower levels in the other cell lines in the panel (Fig. 2B).

In addition, all cells were characterized for their ability to be intoxicated by the *B. anthracis* LT system. Cells were exposed to furin-cleavable PA-WT or MMP-cleavable PA-L1, in combination with the PA-pore–specific fusion toxin, FP59. The concentration at which 50% of the cell population displayed an effect (EC_50_) was determined for the PA-WT/FP59 and PA-L1/FP59 combinations. Whereas HT1080, MDA-MB-231, and B8484 cells were sensitive to both PA-WT/FP59 (EC50 = 25, 40, and 600 pM, respectively) and PA-L1/FP59 (EC50 = 25, 300, and 600 pM, respectively), MCF7 cells were not (Figs. 2C and 2D). Studies including uncleavable PA-U7, or PA- or FP59-negative controls, showed that the system is highly selective and requires all components of the system to deliver the payload (Fig. 2D). These findings are consistent with previous reports using this system on a different panel of cell lines (20).

### MMP-Mediated Delivery by PA-L1 of Fluorescently Labeled LF Variants In Vitro

**FIGURE 3.**
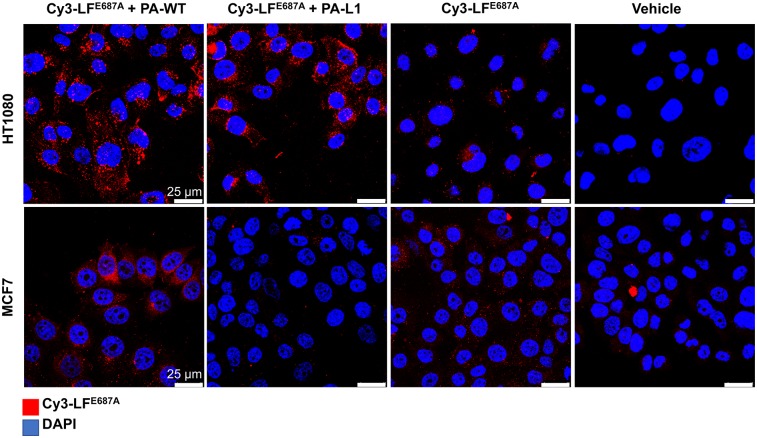
Cy3-LF^E687E^ was delivered to HT1080, but not MCF7, by PA-L1. Images were acquired after fixation and mounted with 4′,6-diamidino-2-phenylindole (DAPI) to highlight nucleus.

LF^E687A^ was fluorescently labeled using a Cy3-dye via a lysine-directed activated ester of Cy3, resulting in Cy3-LF^E687A^. The identity of the resulting construct was determined via absorbance measurements to determine the number of Cy3 moieties per molecule of LF^E687A^ as 6:1. Cy3-LF^E687A^ was taken up in MMP-expressing HT1080 cell lines when combined with the MMP-activatable PA-L1 and when combined with furin-activatable PA-WT. Negligible uptake was observed when no PA was added (Fig. 3). In contrast, only negligible uptake was observed in MCF7 cells in all of the conditions, indistinguishable from Cy3-LF^E687A^-nonspecific uptake, which was potentially due to macropinocytosis. This finding is in seeming contrast to PA-WT/FP59 treatment’s causing some cytotoxic effects in MCF7 cells, possibly because of the very low amount of FP59 molecules necessary to cause cytotoxicity compared with the detection limit of Cy3-LF^E687A^ uptake in these cells (Fig. 2).

### MMP-Mediated Delivery by PA-L1 of ^111^In-LF Variants In Vitro

The PA/LF variants were conjugated to bifunctional chelator agents, which allowed radiolabeling using ^111^InCl_3_. Both PA-WT^K563C^ and LFn-cys, containing an engineered cysteine site that does not interfere with epitope binding, were conjugated to the bifunctional metal ion chelator maleimide-DTPA. PA-L1 and LF^E687A^ were conjugated using lysine-directed chemistry via *p*-SCN-Bn-DTPA. Analysis by mass spectroscopy of the starting products and DTPA-modified version showed that PA-WT^K563C^ and LFn-cys incorporated just 1 DTPA moiety, whereas PA-L1 and LF^E687A^ were conjugated to 2–8 DTPA moieties (Supplemental Fig. 1B). Quality control using instant thin-layer chromatography showed incorporation yields and radiochemical purities of more than 95% (Supplemental Fig. 5). The molar activity of the radiolabeled compounds was 8–15, 8–15, 10, and 5 MBq/nmol for ^111^In-PA-WT^K563C^, ^111^In-PA-L1, ^111^In-LF^E687A^, and ^111^In-LFn, respectively, for in vitro studies. The molar activity of all compounds for in vivo studies is as indicated. All labeled compounds demonstrated satisfactory stability in mouse serum, although a small degree of transchelation could be observed (Supplemental Fig. 11), attributable to transferrin or albumin.

**FIGURE 4.**
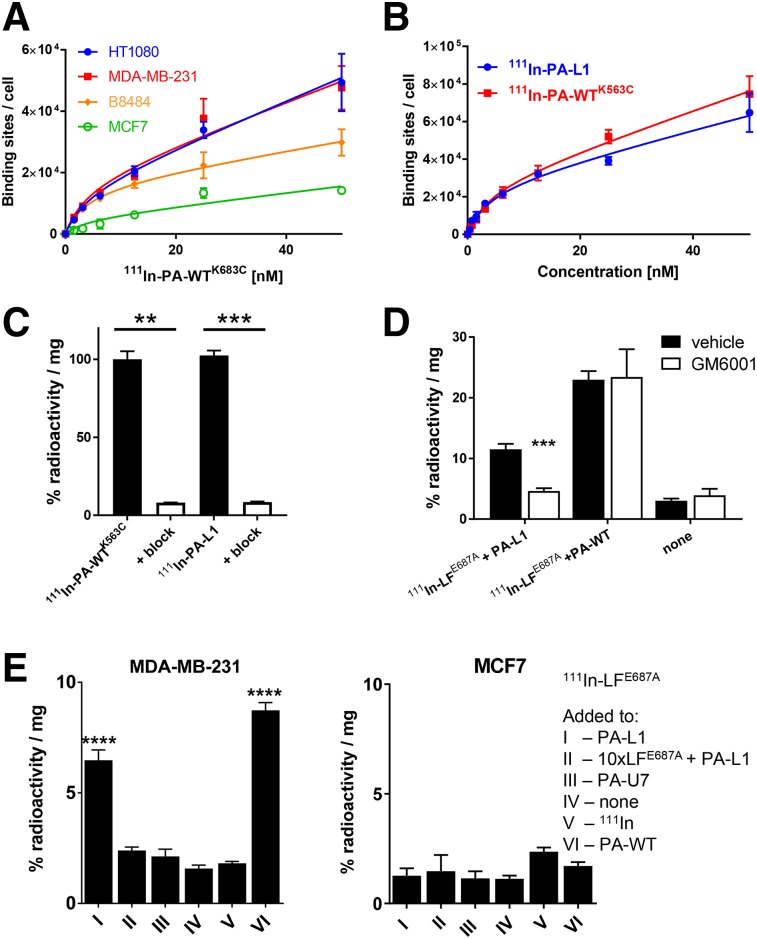
In vitro cell uptake of ^111^In-PA-L1, ^111^In-PA-WT^K563C^, and ^111^In-LF^E687A^. (A) Saturation binding assays using ^111^In-PA-WT^K563C^ determined affinity for and amount of PA binding sites on cells. (B) Binding of ^111^In-PA-WT^K563C^ to cells was not significantly different from ^111^In-PA-WT. (C) Uptake of ^111^In-PA-WT^K563C^ and ^111^In-PA-L1 in MDA-MB-231 cells could be blocked by 100-fold excess of cold unlabeled PA-WT or PA-L1. (D) PA-L1–mediated uptake of ^111^In-LF^E687A^ in MDA-MB-231 cells could be significantly reduced by exposure of cells to broad-spectrum MMP inhibitor GM6001. (E) PA-L1 allowed uptake of ^111^In-LF^E687A^ in MDA-MB-231 cells but not in MCF7 cells. **P < 0.01. ***P < 0.001. ****P < 0.0001.

The radiolabeled versions of PA and LF allowed quantification of their interactions with the cell panel. Saturation binding assays using ^111^In-labeled PA-WT^K563C^ showed various levels of binding sites (∼10–40 × 10^3^; Fig. 4A), which is in line with the relative expression levels of CMG2 detected by Western blot (Fig. 2A). We further showed that binding affinity of PA-WT was not statistically different from that of PA-WT^K563C^, which enabled the use of the latter, which allows site-specific radiolabeling, for imaging purposes (Supplemental Fig. 4C). Affinity of ^111^In-PA-WT^K563C^ for CMG2 and TEM8 was confirmed by a saturation binding assay on CHO cells that were stably transfected to express CMG2 or TEM8 (K_D_ = 4.1 ± 0.6 and 50 ± 9.6 nM, respectively; Supplemental Fig. 6). The overall affinity of ^111^In-PA-WT^K563C^ was measured as K_D_ = 4.0 ± 1.2 nM and was not significantly different in any of the cell lines (*P* = 0.09) (Fig. 4A). Similarly, no significant differences in binding affinity or receptor quantity of ^111^In-PA-WT^K563C^ were observed compared with its MMP-cleavable variant, ^111^In-PA-L1, in MDA-MB-231 cells (Fig. 4B). Using the same experimental parameters, addition of a large excess of unlabeled PA-WT or PA-L1 significantly reduced binding (≤92%, *P* < 0.01), confirming specific binding (Fig. 4C).

In a separate series of measurements, exposure of the panel of cancer cells to a radiolabeled full-length catalytically inactive variant of LF, ^111^In-LF^E678E^, demonstrated the selectivity and specificity of the LT system (Figs. 4D and 4E). A significant reduction in the association of ^111^In-LF^E678E^ with MDA-MB-231 cells after treatment with the broad-spectrum MMP inhibitor GM6001 demonstrated MMP-mediated PA-L1 activation (Fig. 4D; *P* = 0.0003). ^111^In-LF^E678E^ was also taken up by MDA-MB-231 cells when administered in combination with furin-cleavable PA-WT or MMP-cleavable PA-L1 but not in combination with vehicle control or nonactivatable PA-U7 (*P* < 0.0001) (Fig. 4E). Additionally, PA-L1–mediated cell delivery of ^111^In-LF^E678E^ could be significantly reduced by blocking with a 10-fold excess of cold, unlabeled LF^E678E^. No delivery of ^111^In-LF^E678E^ was observed in MCF7 cells under any of the conditions (*P* > 0.05). Similar results were obtained using a labeled LFn, ^111^In-LFn, or using HT1080 and B8484 cells (Supplemental Fig. 7). Saturation binding studies of ^111^In-LF^E687A^ using fixed prepores in MDA-MB-231 cells revealed a binding constant of K_D_ = 0.9 ± 0.09 nM (Supplemental Fig. 8).

**FIGURE 5.**
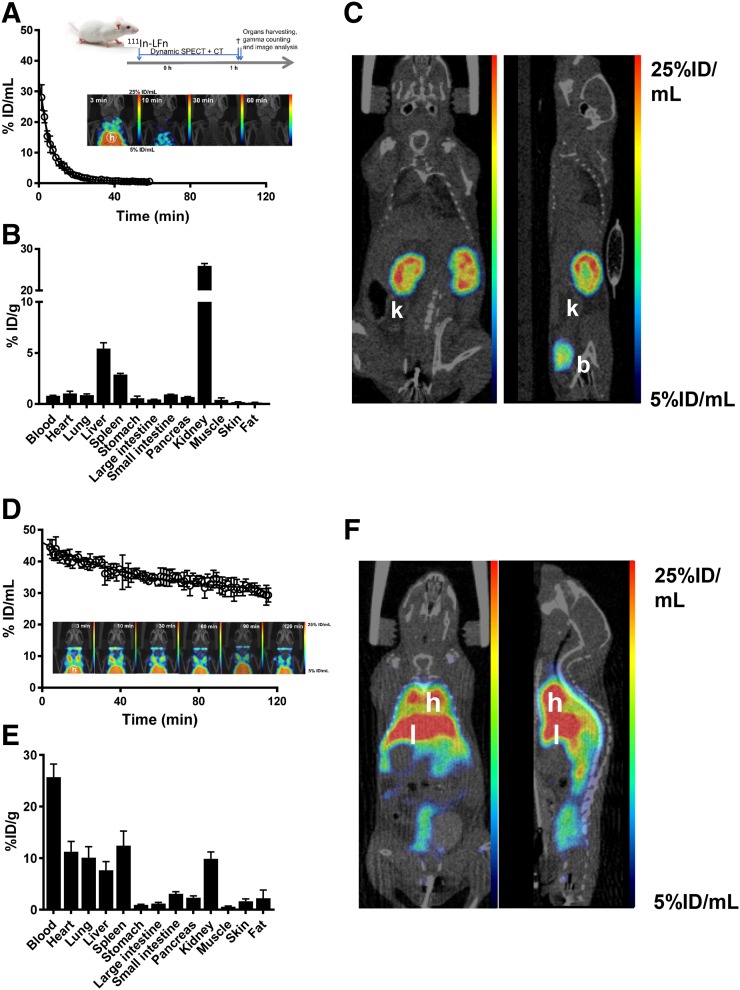
In vivo evaluation of ^111^In-LFn and ^111^In-LF^E687A^ in tumor-naïve SCID mice. (A) Blood clearance of ^111^In-LFn (inset: maximum-intensity projections over time). (B) Ex vivo biodistribution showing uptake of ^111^In-LFn in selected tissues and blood, 1 h after intravenous administration. (C) Representative coronal and sagittal sections of SPECT images acquired 1 h after intravenous administration of ^111^In-LFn. (D) Blood clearance of ^111^In-LF^E687A^ (inset: maximum-intensity projections over time). (E) Ex vivo biodistribution showing uptake of ^111^In-LF^E687A^ in selected tissues and blood, 3 h after intravenous administration. (F) Representative coronal and sagittal sections of SPECT images acquired 3 h after intravenous administration of ^111^In-LF^E687A^. b = bladder; h = heart; k = kidneys; l = liver. Full-sized versions of panels A and D are available in the supplemental materials.

The pharmacokinetic behavior of ^111^In-LFn and ^111^In-LF^E687A^ was compared in vivo. Dynamic SPECT scans were performed after intravenous bolus injection of either ^111^In-LFn or ^111^In-LF^E687A^ in naïve SCID mice (Fig. 5). Imaging data revealed that the truncated ^111^In-LFn presented a biexponential decay in blood with a rapid distribution phase resulting in a fast half-life of 1.8 min, and an elimination phase presenting a slow half-life of 6.0 min. Thirty minutes after injection, only low concentrations of radiotracer in the blood pool were observed (1.1 ± 0.1 %ID/mL). In contrast, the radiolabeled full-length protein, ^111^In-LF^E687A^, presented a significantly slower plasma clearance (*P* < 0.0001) than the truncated protein, maintaining a significant amount in the blood circulation 3 h after administration (35 ± 2.6 %ID/mL). In this case, a monoexponential decay described the radiotracer pharmacokinetic, with a blood half-life of 33 min. Ex vivo biodistribution studies confirmed the in vivo imaging results, revealing low blood signal, yet high kidney uptake (29 ± 1.0 %ID/g), after administration of ^111^In-LFn, possibly because of glomerular filtration (Supplemental Table 1).

### Uptake of ^111^In-PA-L1 and ^111^In-PA-WT^K563C^ by Tumor Tissue In Vivo

**FIGURE 6.**
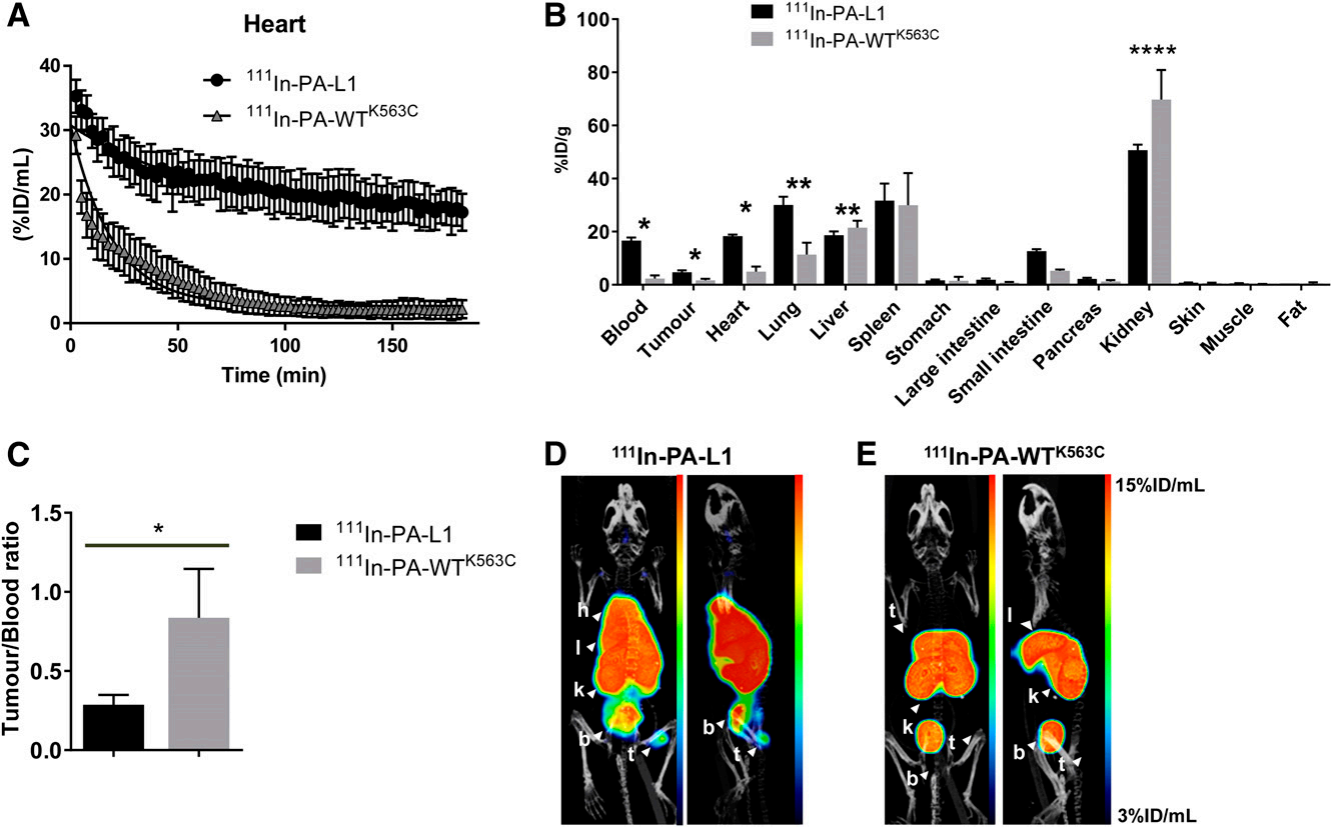
In vivo evaluation of ^111^In-PA-L1 and ^111^In-PA-WT^K563C^ in MDA-MB-231 tumor-bearing SCID mice. (A) Blood clearance of ^111^In-PA-L1 and ^111^In-PA-WT^K563C^. (B) Ex vivo biodistribution showing uptake of ^111^In-PA-L1 or ^111^In-PA-WT^K563C^ in selected tissues and blood, 3 h after intravenous administration. (C) Tumor-to-blood ratios determined from B. (D and E) Representative coronal and sagittal maximum-intensity projections of SPECT/CT images, 3 h after administration of ^111^In-PA-L1 or ^111^In-PA-WT^K563C^. *P < 0.05. **** *P* < 0.0001. b = bladder; h = heart; k = kidneys; l = liver; t = tumor.

The pharmacokinetics of radiolabeled furin–cleavable PA-WT^K563C^ and MMP-cleavable PA-L1 were also compared, with a view to optimizing the time of injection in a ^111^In-LF pretargeting system. Dynamic SPECT imaging after intravenous injection of ^111^In-PA-L1 or ^111^In-PA-WT^K563C^ in MDA-MB-231 tumor–bearing mice revealed a distinct pharmacokinetic profile for ^111^In-PA-WT^K563C^ compared with ^111^In-PA-L1 (Fig. 6A). Both presented monoexponential decay, with blood half-lives of 13.5 and 40.9 min, respectively. SPECT/CT imaging and ex vivo biodistribution showed a significantly increased uptake of ^111^In-PA-L1 (5.7 ± 1.7 %ID/g) in MDA-MB-231 tumor tissue, compared with ^111^In-PA-WT^K563C^ (1.7 ± 0.52 %ID/g; *P* < 0.05), at 3 h after administration of the labeled compound (Figs. 6B–6E; Supplemental Table 2). Contrastingly, tumor-to-blood ratios were higher for ^111^In-PA-WT^K563C^ than for ^111^In-PA-L1 (*P* < 0.05). All in vivo SPECT and ex vivo biodistribution results were confirmed by autoradiography (Supplemental Fig. 9).

### MMP-Mediated Delivery by PA-L1 of ^111^In-LF Variants to Tumor Tissue In Vivo

**FIGURE 7.**
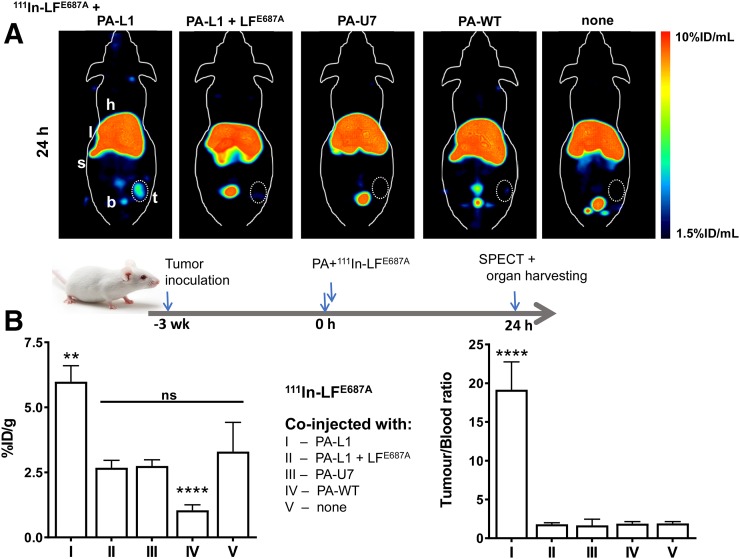
(A) Representative coronal maximum-intensity projections of SPECT images of MDA-MB-231 tumor xenograft–bearing SCID mice, 24 h after administration of ^111^In-LF^E687A^, alone or in combination with (left to right) PA-L1, PA-L1 and excess of unlabeled LF^E687A^, PA-U7, PA-WT, or no PA protein. Tumors are encircled (B) Ex vivo biodistribution showing tumor uptake of ^111^In-LF^E687A^ in different experimental groups. b = bladder; h = heart; l = liver; s = spleen; t = tumor. **P < 0.01. ****P < 0.0001.

On the basis of its pharmacokinetic profile, ^111^In-LF^E687A^ was considered to be the more appropriate variant to image MMP-mediated PA-L1 pore formation in tumor tissue in vivo, given the extremely fast clearance of ^111^In-LFn. In addition, the slow but steady increase in tumor uptake of PA-L1 over time indicated that a coinjection of PA-L1 with ^111^In-LF^E687A^ would be advantageous. The delivery of ^111^In-LF^E678E^ by PA-L1 was evaluated in mice bearing MDA-MB-231 xenografts (Fig. 7). Full ex vivo biodistribution data are summarized in Supplemental Table 3. SPECT/CT images and ex vivo biodistribution analysis at 24 h after injection revealed significantly increased delivery by PA-L1 of ^111^In-LF^E678E^ to tumor tissue (5.98 ± 0.62 %ID/g), compared with control animals that were not coinjected with PA-L1 (group V, 3.30 ± 1.12 %ID/g; *P* = 0.0017), indicating PA-L1–specific delivery. Imaging data at 3 h after injection showed similar results (Supplemental Fig. 10). When animals were also administered noncleavable PA-U7, instead of PA-L1, tumor uptake was significantly reduced (group III, 2.74 ± 0.24; *P* = 0.0004), indicating MMP activity–specific targeting of PA-L1 in vivo. Additionally, when the interaction between PA-L1 pores or prepores and ^111^In-LF^E678E^ was blocked by coadministration of an excess of unlabeled LF^E678E^, tumor uptake was significantly reduced to nonspecific uptake levels (group II, 2.68 ± 0.28 %ID/g; *P* = 0.0003). ^111^In-LF^E678E^ delivery to tumor tissue was significantly lower when coadministered with furin-cleavable PA-WT (group IV, 1.05 ± 0.21 %ID/g; *P* < 0.0001). This finding was in line with the rapid plasma clearance, activation by proteases in plasma (21), and lower tumor uptake of PA-WT. Kidney uptake was also affected by this effect (7.02 ± 2.15 vs. 6.31 ± 0.32 %ID/g, respectively; *P* < 0.0001). Contrastingly, PA-WT–mediated delivery of ^111^In-LF^E678E^ resulted in significantly higher delivery to spleen (29.84 ± 16.10 vs. 126.25 ± 42.18 %ID/g, respectively; *P* < 0.0001), again consistent with PA-WT cleavage and activation in blood plasma.

Taken together, our results indicate the selective delivery to tumor tissue of ^111^In-LF^E678E^ by PA-L1 via binding to anthrax receptors, MMP-activation of PA-L1, and LF binding to its de novo receptor site on PA pores.

## DISCUSSION

Molecular imaging is a promising method to visualize the clinical function of MMPs in a wide variety of diseases such as cancer, atherosclerosis, stroke, arthritis, periodontal disease, multiple sclerosis, and liver fibrosis. Here, we demonstrate a novel concept, using an MMP-activated pretargeting approach, based on the *B. anthracis* LT system. Previous work by Liu et al. showed in detail that PA-L1, an MMP-cleavable PA variant, allows for selective delivery of LF and related fusion toxins such as FP59 to tumor tissue in vivo for therapeutic gain (22). Here, we exploited this system to develop a molecular imaging system using a radiolabeled form of LF.

The PA-L1/LF system used here is distinctly different from any previously reported MMP imaging methodology. An advantage is that the system does not rely on binding to an extracellular, potentially nonpermanent epitope but rather depends on the uptake of radiolabeled ^111^In-LF^E687A^ by cells. Notably, the LT system has the distinct additional advantage that on cleavage of PA-L1 by MMPs, the PA pore is capable of delivering multiple radiolabeled copies of ^111^In-LF^E687A^ to the cytoplasm of tumor cells.

Here, we have demonstrated in a panel of cancer cell lines that MMP-activated PA-L1–mediated delivery of labeled LF variants enables selective visualization and measurement of MMP activity levels in tumor tissue in vivo. We performed detailed characterization of the system and demonstrated selectivity for anthrax receptors, mainly CMG2, high affinity of radiolabeled LF for PA-L1 pores in cells, and selective, specific delivery of ^111^In-LF^E687A^ to cancer cells in vitro and in vivo. PA-L1–mediated ^111^In-LF^E687A^ delivery to cells and tumors was blockable with an excess of LF or could be reduced by replacing PA-L1 with PA-U7, PA-WT, or vehicle only, indicating the PA-L1 and thus MMP-dependent cleavage of this interaction.

Some limitations of the PA-L1/^111^In-LF^E687A^ system to visualize MMP activity are the inherent uptake of ^111^In-LF^E687A^ in liver and spleen, as is possibly due to ER-mediated uptake. Its pharmacokinetics were also rather slow, necessitating SPECT imaging 24 h after intravenous administration. Imaging at earlier time points after injection (we evaluated a 3-h time point) did not show the necessary tumor-to-background ratios compared with the longer interval. In contrast, the smaller size of ^111^In-LFn resulted in renal clearance and therefore negligible liver uptake. ^111^In-LFn performed equally well in in vitro assays but, in vivo, was cleared from the blood so fast that PA-L1–mediated tumor uptake could not be observed (Supplemental Table 4). It may be possible that variants of intermediate size may be cleared more slowly yet are still renally cleared, in analogy with radiolabeled antibody fragments and derivatives, and thus will result in more optimal imaging characteristics (23). It is also notable that because of the complexity of the MMP-activated LT system, the use of a large number of control conditions was required to demonstrate the specificity of the approach. In addition, the immunogenicity of DTPA-LF^E687A^ and DTPA-LFn was not studied in the current work. However, it is known that nonmodified LF, when injected in the bloodstream, induces formation of neutralizing antibodies, resulting in protective immunity (24,25). Finally, the specificity of the PA-L1 protein variant to be cleaved and therefore activated by proteases other than its intended targets (MMP2, MMP9, and MMP14) and deliver ^111^In-LF^E687A^ to tumor cells was not evaluated here. The inherent overlap in specificity of all MMP proteins toward their substrates means that no single substrate will be specific for any given individual MMP. Similar to other proteases, MMP substrate specificity is guided by positional preferences of the residues on both the N terminus and the C terminus from the scissile bond. The substrate of MMPs contain a canonical P-X-X-↓L motif, with ↓ denoting the scissile bond (26). Depending on the nature of the other amino acids, the resulting sequence can be cleaved by all MMPs or show a preference for some individual MMPs or groups. This high level of substrate cleavage redundancy explains the functional overlap observed among members of the MMP family.

Additionally, our study showed the pharmacokinetics and tissue-binding properties of radiolabeled PA-WT^K563C^ and LF. As expected, uptake of ^111^In-PA-WT^K563C^ was observed in organs where the presence of CMG2 receptors has been previously described (27,28). Its fast clearance from the blood pool, when compared with that of ^111^In-PA-L1, indicates that this molecule may be cleaved by proteases in the blood and that part of the observed organ uptake corresponds to labeled PA fragments rather than the interaction of the intact PA-WT^K563C^ with its receptors (21). Renal uptake of ^111^In-LF^E687A^ in animals that also were exposed to PA-WT were similar to unspecific uptake levels. This finding may indicate that the uptake of PA-WT^K563C^ observed in the kidneys was not due to competent PA-WT^K563C^ pore formation. Conversely, mice treated with PA-WT and ^111^In-LF^E687A^ presented higher liver, lung, and spleen uptake of ^111^In, suggesting higher levels of intoxication in these organs. These results suggest that PA-WT, and PA-WT^K563C^, are interacting with their cellular receptors and forming competent pores after having been cleaved, resulting in delivery of ^111^In-LF^E687A^ to these tissues. Although this tool could be used to study LT intoxication, the delivery of labeled LF did not correlate with an earlier report by Liu et al. In this study, it was demonstrated in various cell-type–specific CMG2-null mice, the key tissues responsible for LT lethality are cardiomyocytes and smooth vasculature in the heart (29–31), indicating that targeting and toxicity of LT are not interchangeable.

## CONCLUSION

Taken together, our results indicate that radiolabeled forms of mutated anthrax LT hold promise for noninvasive imaging of MMP activity in tumor tissue.

## DISCLOSURE

This research was supported by CRUK through the Oxford Institute for Radiation Oncology and the CRUK Oxford Centre, the CRUK/EPSRC Imaging Centre in Oxford, and Pancreatic Cancer U.K. Mary-Ann Elvina Xavier is funded by a Science Without Borders (CNPq) grant. Julia Baguña Torres is funded through a grant from the Pancreatic Cancer Research Fund. Thomas Bugge is funded by the NIDCR, NIH Intramural Research Program, and Samantha Hopkins is funded by the NIAID, NIH Intramural Research Program. Mass spectrometry analysis was performed at the Discovery Proteomics Facility (headed by Roman Fischer), which is part of the TDI MS Laboratory led by Professor Benedikt Kessler. No other potential conflict of interest relevant to this article was reported.

## KEY POINTS

**QUESTION:** Radiolabeled engineered anthrax proteins allow detection of matrix metalloproteinases (MMPs) in mouse models of cancer, using SPECT.
**PERTINENT FINDINGS:** Engineered anthrax proteins are activated specifically by MMP activity. A radiolabeled variant of anthrax lethal toxin was selectively delivered to MMP-active xenograft tumors in mice, presenting a 20-fold target-to-background contrast.
**IMPLICATIONS FOR PATIENT CARE:** Imaging overactive MMPs with radiolabeled engineered anthrax proteins can aid in the prognosis of cancer patients and early detection.
