# Supplementary material for: Tumor Imaging Using Radiolabeled Matrix Metalloproteinase–Activated Anthrax Proteins
Source: J Nucl Med. 2019 Oct;60(10):1474–82. doi: 10.2967/jnumed.119.226423 (PMC6785798; doi:10.2967/jnumed.119.226423)
Supplement: Supplementary file 1 [file jnm226423SupplementalData.pdf]

A

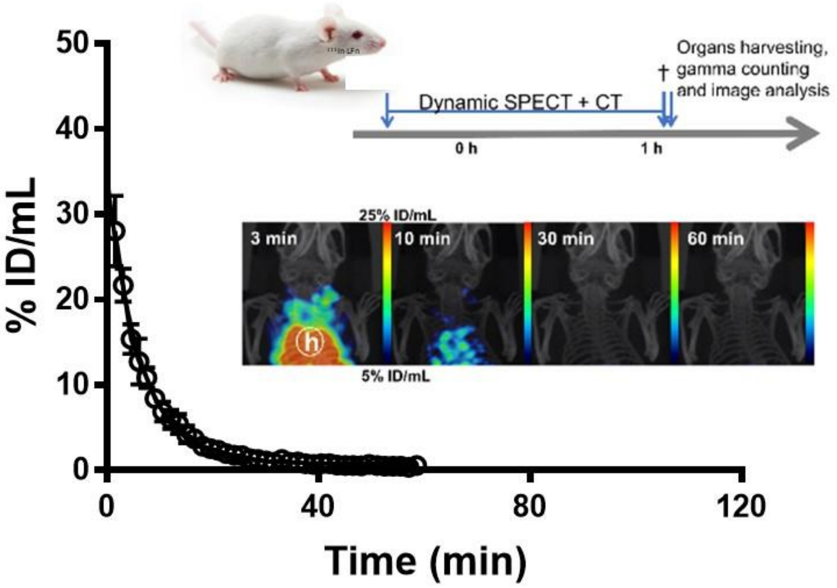

D

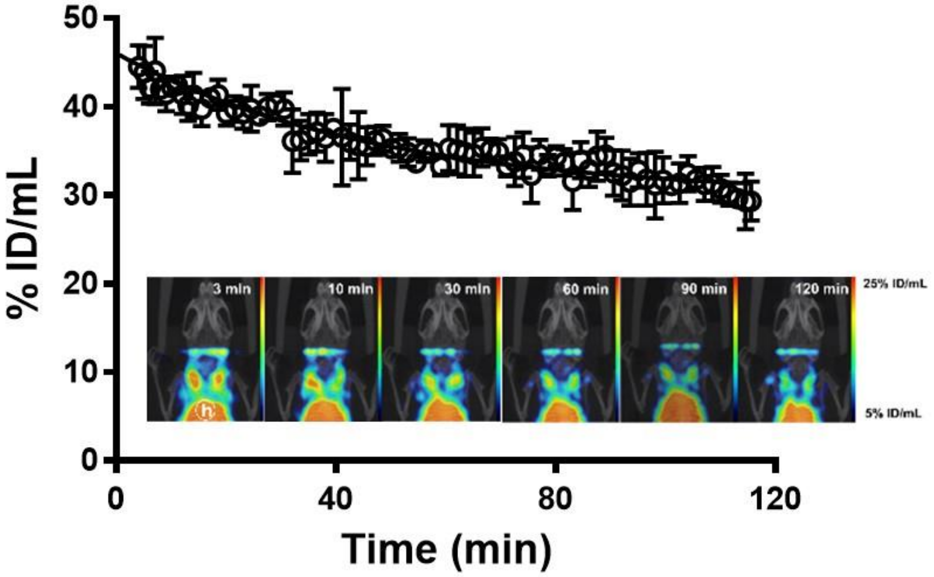

Full-size versions of Figures 5 A and 5D, to show detail.

Supplemental Materials and Methods

- ***Cleavability assays***

The different PA variants were incubated with active recombinant human MMP2 (62 kDa, Sigma, Dorset, UK), recombinant soluble human furin (64 kDa; Abcam, Cambridge, UK), and recombinant soluble human MMP14 (20 kDa; R&D systems, Abindgdon, UK). rhMMP14 (40 µg/mL) was first activated by addition of rhFurin (0.86 µg/mL) for 90 min in activation buffer (50 mM Tris pH 9.0, 1 mM $CaCl_2$, 0.5% (v/v) Brij-35) at 37°C. Cleavage by MMPs was performed in 50 mM HEPES (pH 7.5), 10 mM $CaCl_2$, 200 mm NaCl, 0.05% (v/v) Brij-35, and 50 µM $ZnSO_4$. Reaction mixtures of 50 µL containing 10 µg of PA proteins were incubated with rhMMP2, rhMMP14 or rhFurin at 37°C for 16 h in assay buffer (50 mM Tris, 3 mM $CaCl_2$, 1 µM $ZnCl_2$, pH 8.5). PA MMP2 cleavage reaction mixture comprised of 25 mM HEPES (pH 7.4), 150 mM NaCl, 0.2 mM EDTA, 0.2 mM EGTA, 100 µg/mL ovalbumin, 1.0 mM $CaCl_2$, and 1.0 mM $MgCl_2$. Sample aliquots were then separated by SDS-PAGE using 4–12% gradient Tris-glycine gel (Novex, San Diego, CA). Additionally, PA-L1 and PA-U7 cleavage was analysed by intact protein Mass spectrometry.

- ***Mass spectroscopy***

Reverse-phase chromatography was performed in-line prior to mass spectrometry using an Agilent 1290 HPLC system equipped with a Zorbax 300SB-C3 column (4.6x150 mm), using 0.1% formic acid in ultrahigh purity water (solvent A) and 0.1% formic acid in methanol (solvent B) as the mobile phase, using gradient elution as follows: 95% A and 5% B and a flow rate of 0.6 ml/min for 2 min followed by a linear gradient 95% B over 5 min. Protein intact mass was determined using Jetstream electrospray ionisation into an Agilent 6550 IMQTof operated in positive-ion mode. The ion source was operated with the capillary voltage at 4000 V, nebulizer pressure at 20 psig, drying gas at 150 °C, and drying gas flow rate at 5 L/min and sheath gas 12 L/min. Data analysis was performed using the MassHunter Qualitative Analysis Version B.07.00 Agilent software. The deconvoluted mass for each protein was compared with the theoretical mass of the proteins.

- ***In vitro infection of cells by LT***

Cells were harvested using trypsin (Sigma, Dorset, UK) and seeded in 96-well plates ($3x10^3$ - $5x10^3$ cells/200 µL) and plates were incubated at 37°C and 5% CO_2. At 80% confluence, 100 µL of the conditioned media was replaced with PA-WT, PA-L1 or PA-U7 at different concentrations combined with FP59, LFE687A or LFn. At different time points MTT (3-(4, 5-dimethylthiazol-2-yl)-2, 5 diphenyltetrazolium bromide, Sigma, Dorset, UK) was added at a final concentration of 0.5 mg/mL and incubated for 2 h at 37°C. Reduced MTT salt was dissolved using dimethyl sulfoxide (DMSO, Sigma) after removal of conditioned media. Absorbance at 495 nm was measured and considered as a signal of cell viability. To investigate PA activation by MMPs, cells were washed three times with serum-free media and incubated with the broad spectrum MMP inhibitor GM6001 (30 µM; Abcam, Cambridge, UK) for 30 min prior to adding LT components. After incubation for 6 h at 37°C, cells were washed 3 times and a fresh complete cell culture media was added. After a further 48-hour incubation, cell viability was determined by the MTT assay.

- ***Zymography***

Gelatin zymography analysis was used to gauge the level of MMP2 and MMP9 secretion of cells. Conditioned medium was harvested from the various cell lines, after concentrating the secretome by centrifugation over a micro-filter with 30 kDa MW cut-off, and gelatin zymography was performed as previously described (*30*). Additionally, whole cell lysates were prepared using RIPA buffer, and SDS-PAGE followed by Western blot was used to compare the extent of MMP14 and CMG2 expression between the different cell lines, using β-actin as a loading control. Results were corroborated by immunofluorescence.

HT1080, MDA-MB-231, B8484 or MCF7 cells were seeded onto 6-well plates (2.5×10^5 cells/well) and incubated overnight at 37°C, 5% CO_2. When cells reached 70-80% confluence, they were washed twice with serum-free culture medium and incubated again with the same media type. After a further 16 h, conditioned medium was harvested and cleared by centrifugation at 8,800 x g for 10 min at 4°C and either used immediately, or stored at -80°C until use. Samples were concentrated using 10 kDa centrifugal filter (Millipore) at 17,300 x g, 4°C for 10 min. Five microliters of concentrated sample was vortexed with Tris-Glycine SDS sample buffer and loaded in a 10% gelatin gel (Novex). After electrophoresis, the gel was washed in dH_2O and incubated with renaturing buffer (Novex) for 30 min at room temperature under gentle agitation. Subsequently, developing buffer (Novex) was used to equilibrate the gel for 30 min, after which the gel was incubated at 37°C for 30 h with fresh developing buffer.

In control experiments, the broad-spectrum MMP inhibitor GM6001 (30 µM; Abcam, Cambridge, UK) was added in the developing buffer. Gels were stained with SimplyBlue SafeStain solution (Thermo Scientific) for 3 h to visualise digested gelatin as non-stained bands in the gel.

- ***Western blot***

Cells were harvested using ice cold PBS and centrifuged at 16,500 x g at 4°C for 5 min. Cell lysates were prepared incubating cell pellet for 30 min on ice using radioimmunoprecipitation assay buffer (RIPA; 50 mM Tris-HCl, pH 8.0, 150 mM NaCl, 1% Triton X-100, 0.5% sodium deoxycholate; Sigma, Dorset, UK) and protease inhibitor cocktail (Sigma, Dorset, UK). Cell suspensions were sonicated three times at 50% amplitude using a 30 sec on/ 10 sec off cycle. Homogenized cells lysates were cleared by centrifugation at 16,500 x g at 4°C for 20 min. Protein concentration of the supernatant was determined using the colorimetric bicinchoninic acid assay (BCA) (Sigma, Dorset, UK). Protein samples separated in a pre-cast SDS-PAGE gel (ThermoFisher Scientific, Paisley, UK). Protein was transferred to polyvinylidene difluoride membrane using a tank blotting system (BioRad, Watford, UK). Membranes were blocked using TBS buffer (Santa Cruz Biotechnology, Wembley, UK) with 0.5% [v/v] Tween 20 and 5% [w/v] skim milk (both supplied by Sigma, Dorset, UK). Blots were incubated with appropriate primary antibodies in blocking solution overnight at 4°C. Rabbit primary antibodies anti-β-actin, anti-MMP14, anti-TEM8 and anti-integrin beta 1 (all provided by Abcam, Cambridge, UK) were used at 1:2,000 dilution. Goat anti-CMG2 (1:2,000; R&Dsystems, Abindgdom, UK) and mouse anti-furin (1:100; Santa Cruz Biotechnology, Wembley, UK) were also used as primary antibodies. The blots were washed three times for 10 min each in TBS with 0.5% v/v Tween 20 (Sigma, Dorset, UK) and incubated with the appropriate horseradish peroxidase secondary antibodies (BioRad, Watford, UK) for 1 h at room temperature. The blots were washed as above and then incubated for 5 min in enhanced chemiluminescence reagents (ThermoFisher Scientific, Paisley, UK) and exposed to x-ray film (Kodak, New York, UK).

- ***Immunocytochemistry***

To visualise MMP14 and CMG2 on cell surfaces, cells were seeded on gelatin-coated coverslips in 24 well plates and left overnight to adhere. Cells were washed twice with ice-cold PBS and stained with rabbit anti-MMP14 (Abcam, ab51074, EP1264Y, 1:2000) and goat anti-

CMG2 (R&D, AF2940, 1:2000) for 1 h at 4°C. The cells were then washed three times with ice-cold PBS and fixed in 4% PFA for 10 min at room temperature, incubated in PBS containing 5% donkey serum for 1 h at room temperature, and then stained with donkey anti-goat IgG Alexa fluor 488 (Life Technologies, 1:2000) and donkey anti-rabbit IgG Alexa fluor 594 (Life Technologies, 1:2000) for 1 h at room temperature. Following three washes with PBS, coverslips were mounted using Vectashield containing DAPI (Vectorlabs). The slides were imaged using a 63X oil objective on a Leica TCS SP8 (Leica, Milton Keynes, UK) laser scanning confocal microscope

- ***Fluorescent labelling of LF^{E687A}***

LF^{E687A} molecules were labelled with Cy3-NHS (GE Healthcare, Amersham, UK). The bioconjugation reaction was performed according to the manufacturer instruction using 200 µg of protein and a 10-fold molar excess Cy3-NHS in sodium carbonate buffer (pH 9.5). The reaction mixture was incubated at 25°C for 2 h under continuous agitation at 550 rpm using a ThermoMixer C (Eppendorf, Stevenage, UK). Molecules of non-conjugated dye were separated from fluorescent molecules, by gel filtration chromatography using Sephadex G-50 (Sigma, Dorset, UK) in mini-columns (height 90.0 x diameter 5.0 mm). Protein samples were eluted using 100 µL PBS for each fraction. The absorbance of these fractions was measured at 280 nm and 550 nm for samples containing Cy3 wavelength using nanodrop system (ThermoFisher Scientific, Paisley, UK). The ratio of dye molecule per protein was determined according to the manufacturer.

- ***Cell uptake of Cy3- LF^{E687A}***

Confocal microscopy was used to visualise the subcellular localization of Cy3–LF^{E687A} in HT1080 and MCF7 cells with or without PA-WT or PA-L1. Sterile round glass coverslips (ThermoFisher Scientific, Paisley, UK) was dipped in 70% ethanol and placed in the bottom of the 24-well plates. Aliquots of 50×10^3 cells/mL were seeded and left overnight to allow cell adherence. Conditioned media prepared with 300 ng/mL Cy3-LF^{E687A} in the presence or absence of 1200 ng/mL PA-WT or PA-L1 was incubated for 3 h at 37°C. After treatment, the cells were washed 3 times with cold PBS and fixed for 10 min at room temperature with 4.0% v/v paraformaldehyde (Sigma) in PBS. Coverslips were gently placed on glass slides (Thermo Fisher) containing VECTASHIELD mounting media and DAPI (Vector Laboratories, Peterborough, UK) for visualisation of cell nuclei. The slides were imaged using 63X oil

objective using a Leica TCS SP8 (Leica, Milton Keynes, UK) laser scanning confocal microscope.

- ***Radiolabelling***

All radiolabelling was performed using Chelex 100 (BioRad, Watford, UK) treated nanopure water to generate buffers. Diethylenetriaminepentaacetic acid maleimide (maleimide-DTPA, CheMatech, Dijon, FR) was conjugated to LFn-cys or PA-WT^{K563C} and isothiocyanatobenzyl-diethylenetriamine-pentaacetic acid (p-SCN-Bn-DTPA, Macrocyclics, Texas, USA) was used to modify PA-L1 or LF^{E687A}. A tenfold molar excess of chelator was used to modify 200 µg of protein in 100 µL PBS buffer (pH 7.4) or 100 µL Sodium Carbonate buffer (pH 9.5). The reaction mixtures were incubated for 2 h under agitation (550 rpm) at 25°C in a Thermomixer C (Eppendorf, Stevenage, UK). Free chelator molecules were removed from protein-bound DTPA by size exclusion chromatography using a mini-column (90 x 5.0 mm) with Sephadex G–25 resin or Sephadex G–50 (Sigma, Dorset, UK) eluted with PBS. Absorbance at 280 nm was determined for each fraction eluted using nanodrop system (ThermoFisher Scientific, Paisley, UK). Pooled fractions of DTPA bound to protein was concentrated to 2 mg/mL. PBS was exchanged to 0.5 M MES Buffer pH 5.5 using centrifugal filters (Millipore, Watford, UK) with three centrifugation steps at 17,500 x g (4°C).

Radioactivity was measured using a dose calibrator (Capintec, New Jersey, USA) and ^{111}In chloride (20 MBq, in 0.02 M HCl, Mallinckrodt) was added to 20 µg of DTPA-PA-L1, DTPA-PA-WT^{K563C}, DTPA-LFn or DTPA-LF^{E687A}. The reaction mixture was incubated at room temperature for 2 h. The radiolabelling purity of ^{111}In-LFn, ^{111}In-LF^{E687A}, ^{111}In-PA-L1 or ^{111}In-PA-WT^{K563C} samples was determined by instant thin-layer chromatography (iTLC) using 0.1 M Citrate Buffer at pH 5.5 (Sigma, UK) as the mobile phase and strips of glass microfiber chromatography paper (Agilent, USA) impregnated with silicic acid as the stationary phase.

- **Stability of radiolabelled LT components**

To mimic tracer stability in vivo, all LT radiolabelled components were incubated with commercially available mouse serum (Sigma, Dorset, UK). After radiolabelling reaction, compounds (1 MBq in 10 uL) were incubated in 500 µL of mouse serum at 37°C under agitation (300 rpm, Eppendorf, UK). At each time point, 5 µL of the mixture was removed, added to sample buffer (without denaturing agent), and heated (10 min, 70°C) before being analysed by SDS-PAGE. Samples were onto a 4-12% Bis-Tris protein gel (170 V, 40 min).

The gel was then exposed to Super Resolution Phosphor screens (Type SR, PerkinElmer) and developed on the Cyclone Plus Storage Phosphor system (Perkim Elmer). For reference, the gel was stained with coomassie stain (SimplyBlue™) to correlate each radioactive band to specific protein weights. To evaluate in vitro stability mimicking cell-based assays, tracer stability was also analysed in 500 µL of PBS and incubated at 37°C (at 300 rpm) and analysed by iTLC as described above.

- ***In vitro* binding and uptake studies**

Radioligand saturation cell binding experiments were carried out to determine the apparent affinity constant (K_d) and maximum number of binding sites (B_{max}) on cancer cells for ^{111}In-PA-WT^{K563C} or ^{111}In-PA-L1. Briefly, 50×10^3 cells/well were plated and incubated overnight in a 24 well plate at 37°C, 5% CO_2. Adherent cells were then washed twice with PBS and fixed for 10 min at room temperature with 4.0% v/v paraformaldehyde (Sigma, Dorset, UK) in PBS. The fixative agent was removed and cells were washed two times with PBS and incubated with blocking buffer consisting of 10 % FBS (ThermoFisher Scientific, Paisley, UK) in PBS for 1 h at room temperature. The total number of cells was determined from a different plate that was not fixed. After trypsinisation live cells were counted using an automated cell counter *Countess II* and Trypan blue solution 0.4% [w/v] (both provided by ThermoFisher Scientific, Paisley, UK). Saturation binding assays were performed with increasing concentrations (0–50 nM) of ^{111}In-PA-WT^{K563C} or ^{111}In-PA-L1 in blocking buffer (10 % FBS in PBS) in a total volume of 300 µL for 3h at room temperature. Cells were then washed twice with ice-cold PBS to remove unbound radioactivity and subsequently dissolved in 2 M NaOH and measured in an automated gamma counter 2470 WIZARD-2 (PerkinElmer). K_D and B_{max} values were obtained using non-linear regression using a one-site saturation binding model (Total Binding = B_{max}*Free/(K_D+Free) + NS*Free + Background), using GraphPad Prism.

To assess the affinity of ^{111}In-LF^{E687A} to PA (pre)pores, CHO-CMG2 cells were plated at a density of 250×10^3 cells/well in 24-well culture plates. After complete cell adhesion the medium was replaced with DMEM containing 80 nM of purified PA-U7, PA-L1 or PA-WT and plates were placed at 4°C for 16 h. The medium was removed by aspiration, and the cells were washed gently 3 times with ice cold PBS. DMEM containing various concentrations of ^{111}In-LF^{E687A} in 300 µL was placed on the plates well. Plates were incubated at 4°C for 2 h. The medium was aspirated, and the monolayers were washed twice with ice cold PBS to remove unbound proteins. Cells were solubilized with 0.1 M NaOH and transferred to

individual tubes. The radioactive content of ^{111}In-LF^{E687A} associated with the cells was measured using an automated gamma counter (Perkin Elmer). To confirm the formation of PA63 bound to CHO-CMG2 cell receptors, Western blot analysis were performed using cells that were incubated with either PA-U7, PA-L1 or PA-WT for 16 h at 4°C to ensure that PA63 fragments would remain on the cell surface. The antibody used was ab1992 (Abcam, clone BAP0105, 1:2000).

Cell internalization assays with either 25 nM of ^{111}In-LF^{E687A} or ^{111}In-LFn was performed to evaluate their capacity to interact with PA pores and be delivered to cells after the radiolabelling reaction. First, 50×10^{3} cells were seeded in 24-well plates and incubated for 48 h at 37°C, 5% CO_2. Cells were exposed to either ^{111}In-LFn or ^{111}In-LF^{E687A} in the presence or absence of 80 nM different of PA variants for 3 h at 37°C using 300 µL of conditioned cell media. Blocking experiment with an excess of unlabelled LFn or LF^{E687A} was achieved with a pre-incubation of a 10-fold excess for 30 min prior to addition of radiotracer and 80 nM PA-L1. Cells were then washed twice with ice-cold PBS to remove unbound radioactivity and subsequently dissolved in 0.1 M NaOH and had their radioactivity detected by automated gamma counter 2470 WIZARD^{2} (PerkinElmer). The amount of radioactivity was normalized by the protein content determined by BCA (Sigma, Dorset, UK) assay and used to normalize the values of radioactivity associated with cells.

- ***SPECT/CT imaging***

Tumour xenografts were generated by subcutaneous injection of MDA-MB-231 cell suspensions (5×10^{6} cells) in the hind flank of SCID animals. A single tumour was implanted per animal. Animals were used for further studies when tumour volume exceeded 0.2 mL, as measured by calliper, after randomisation into groups.

Data were acquired in list mode using MILabs acquisition software v7.39. Triple-energy-window based scatter correction was applied for a photon peak window (140-200 keV). All images were reconstructed with MILabs reconstruction software v3.24 on 0.8 mm isotropic 3D voxel grids using dual matrix similarity regulated ordered-subset expectation maximization (dual matrix SROSEM). After reconstruction, the SPECT and corresponding CT data were co-registered and re-sampled to equivalent 200 µm voxel sizes. CT based attenuation correction was applied.

After imaging, mice were euthanised by cervical dislocation and selected organs, tissues and blood were removed. The samples were immediately rinsed with water, blot dried, and transferred into pre-weighed tubes. After weighing the organs, the amount of radioactivity in each tube was measured using a 2480 WIZARD^2 (PerkinElmer). Counts per minute were converted into radioactivity units (MBq) using calibration curves generated from known standards. These values were decay-corrected to the time of injection, and the percentage of the injected dose per gram (%ID/g) of each tissue was calculated. Tumor tissue was stored in 30% sucrose overnight and flash-frozen using dry ice and stored at -80°C until required for further processing.

- ***Autoradiography and H&E***

For autoradiography, slides containing tumor tissue were exposed to a Super Resolution Phosphor screen (Type SR, PerkinElmer) in autoradiography cassettes for 48 h at 4°C. The screen was then digitally developed using the Cyclone Plus Storage Phosphor system (PerkinElmer). The same tissue sections were stained with hematoxylin and eosin (H&E) to characterise tissue morphology, staining for the nuclei and cytoplasm respectively. Briefly, tissue sections were first washed under running water for 5 min and subsequently stained with filtered Mayer's hematoxylin (Sigma-Aldrich) for 2 min. Slides were rinsed in deionised H_20 (ddH2O) for 20 min prior to staining with 1% eosin [v/v] for 10 s. Finally, sections were dehydrated in increasing concentrations of ethanol (70%, 95%, and 100% [v/v] in ddH2O; Fisher Scientific) and cleared in xylene (Fisher Scientific). Slides were mounted using DPX (distyrene, plasticiser, and xylene) mounting medium (Fisher Scientific) and scanned at 20 × magnification with the ScanScope CS system (Aperio, USA). Adjacent sections were also stained for CMG2 with an aim to acquire immunofluorescence micrographs, using a protocol based on immunocytochemistry, as above. H&E and immunofluorescence micrographs were coregistered with autoradiography images using MATLAB-based software developed in-house (MathWorks, UK) with manually chosen landmarks.

- ***Immunohistochemistry on tissue sections***

Adjacent tumor tissue sections were rehydrated in PBS for 10 min, fixed with 4% PFA for 10 min at 4°C, and washed three times in PBS for 5 min at room temperature. Samples were incubated in blocking buffer (5% [v/v] donkey serum in PBS) for 1 h at room temperature. Subsequently, slides were incubated with goat anti-CMG2 (R&D, AF2940, 1:50) overnight at

4°C. After washing 3 times with PBS, slides were incubated for 1 hour with donkey anti-goat IgG AlexaFluor 488 (Life Technologies, 1:500) at room temperature. Finally, slides washed and counterstained with DAPI (1:1,000 in PBS) to visualise nuclei and mounted using cover-slips and Vectashield mounting medium for fluorescence microscopy (Vector Laboratories). The slides were imaged using 63× oil objective using a Leica TCS SP8 (Leica, Milton Keynes, UK) laser scanning confocal microscope.

Supplemental Results

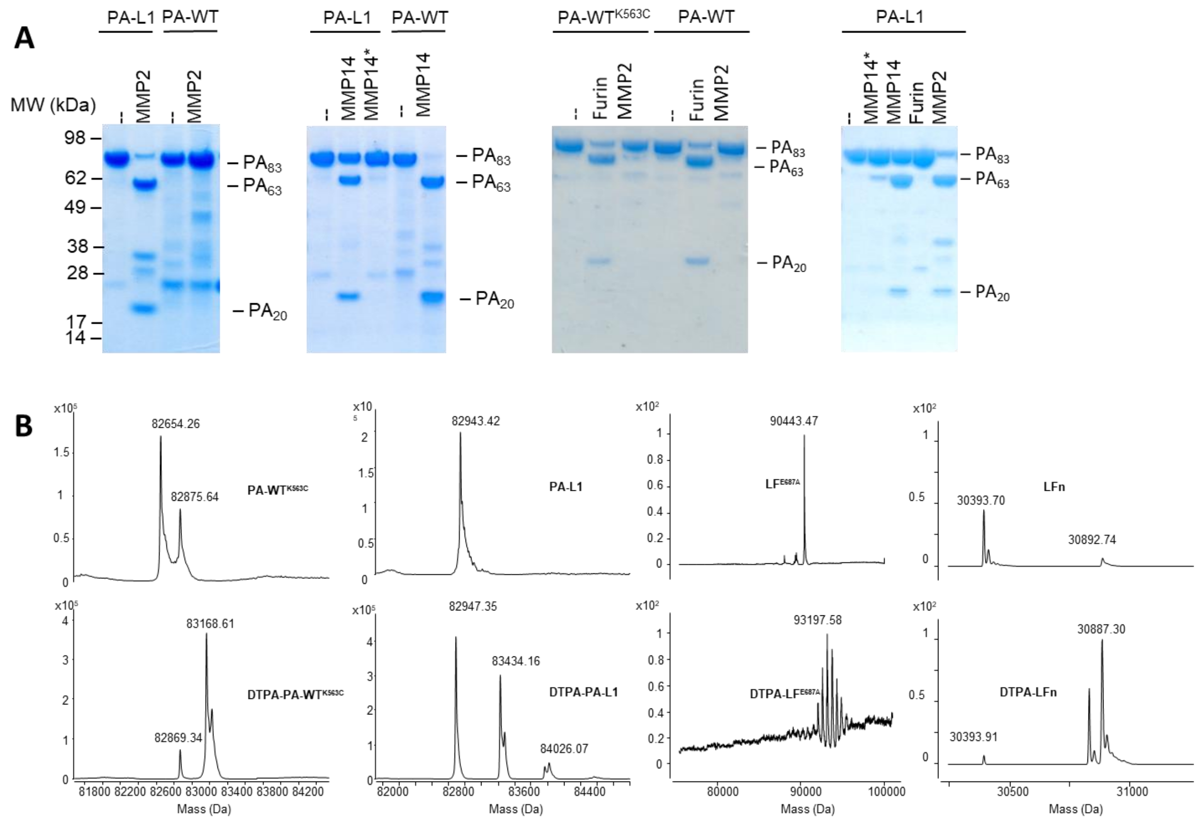

Supplemental Figure 1. Additional *c*haracterisation of LT components. (A) SDS-PAGE analysis of PA variants after incubation with furin or MMPs (2 h at 37°C). - MMP14 not pre-activated with furin (indicated as MMP14*) was used as a control to evaluate the presence of MMP14 bands in the gel.

(B) Mass spectra obtained from intact protein LC-MS for the DTPA-conjugates, as indicated.

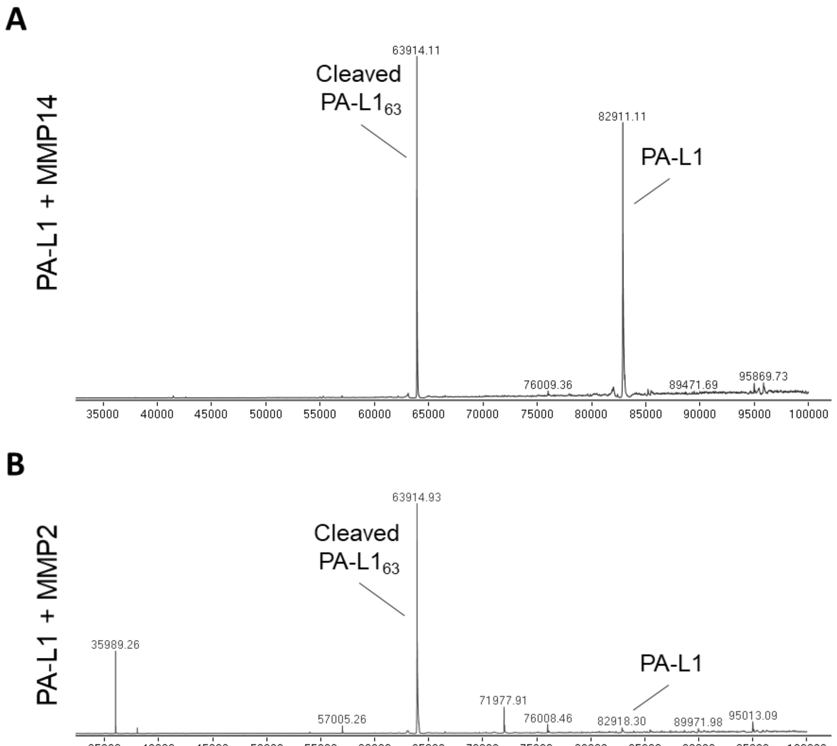

Supplemental Figure 2. MMP cleavage analysis of PA-L1 by LCMS. PA-L1 was exposed to MMP2 or MMP14 for 2 h at 37°C. Mass spectrum analysis demonstrates the cleavage of PA-L1 by both MMP2 and MMP14 at the expected scissile bond.

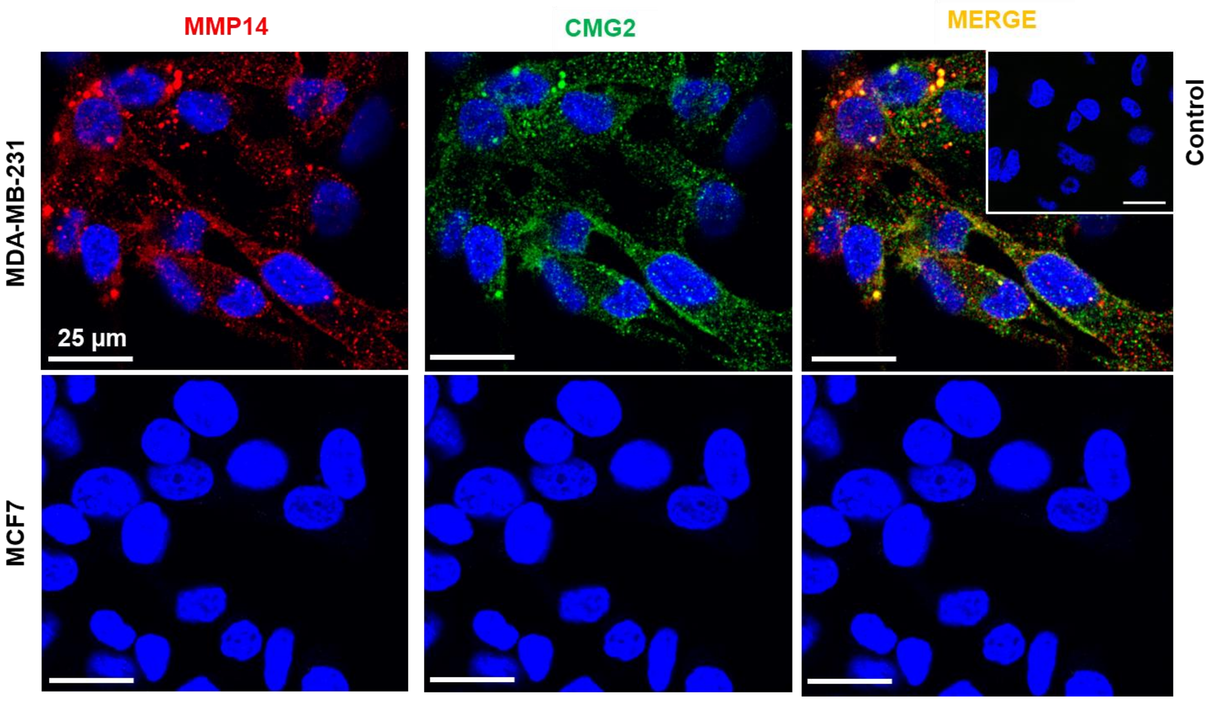

Supplemental Figure 3. Immunofluorescence for MMP14 (red) and CMG2 (green) in MDA-MB-231 or MCF7 cells indicates that MMP14 and CMG2 colocalise (yellow). DAPI (blue) was used to stain the nucleus. Scale bar: 25 µm. The insert shows MDA-MB-231 cell staining with omission of the primary antibodies, to demonstrate the absence of any non-specific staining or autofluorescence signal.

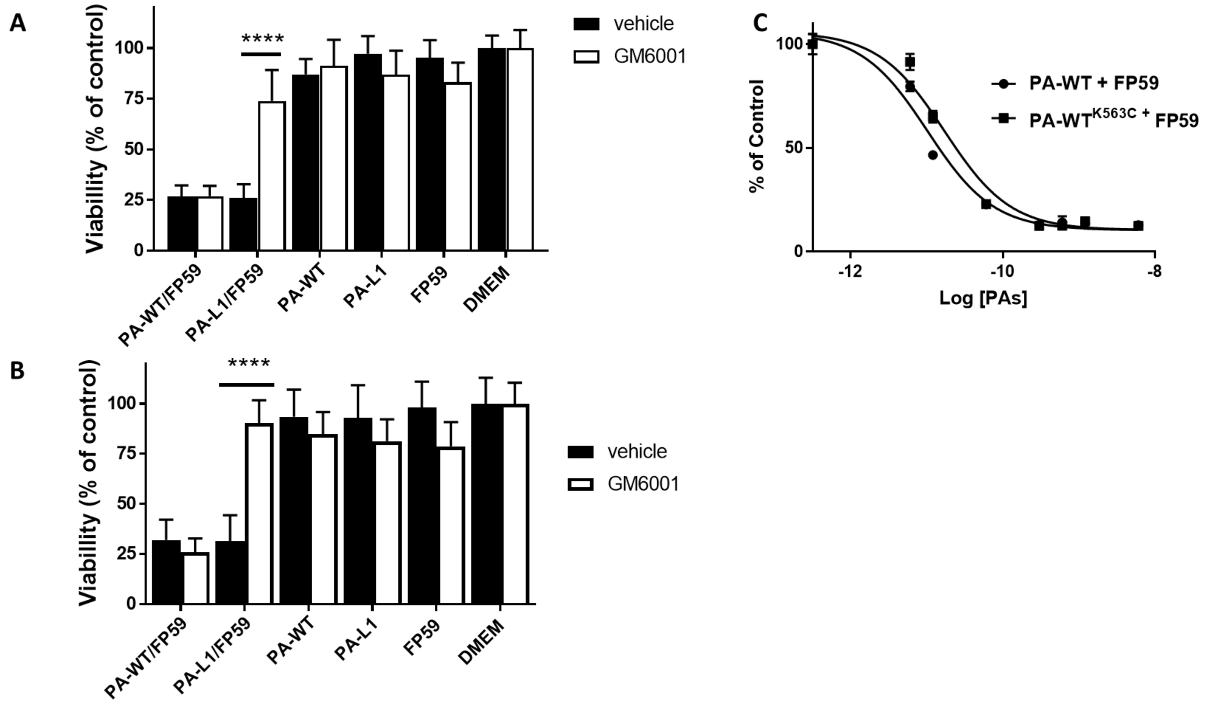

Supplemental Figure 4. Additional MTT assays. (A, B) HT1080 cancer cells were washed before pre-incubating with the broad matrix metalloproteinase (MMP) inhibitor GM6001 for 30 min. Subsequently 300 ng/mL of PA-WT or PA-L1 was added in combination with 50 ng/mL (A) or 1 ng/mL (B) FP59 toxin. After 6 h, the medium containing the toxins and MMP inhibitors was removed, and fresh serum containing medium was added. 48 h after treatment, MTT salt was added to determine cell viability. These results demonstrate that the MMP inhibitor GM6001 selectively rescues cells from intoxication with MMP-activated PA-L1, but not furin-activated PA-WT. (C) Cytotoxic assay using variable concentrations of PA-WT or PA-WT^{K563C} in combination with FP59 in HT1080 cells (log EC50 = 10.99 and 10.77 nM).

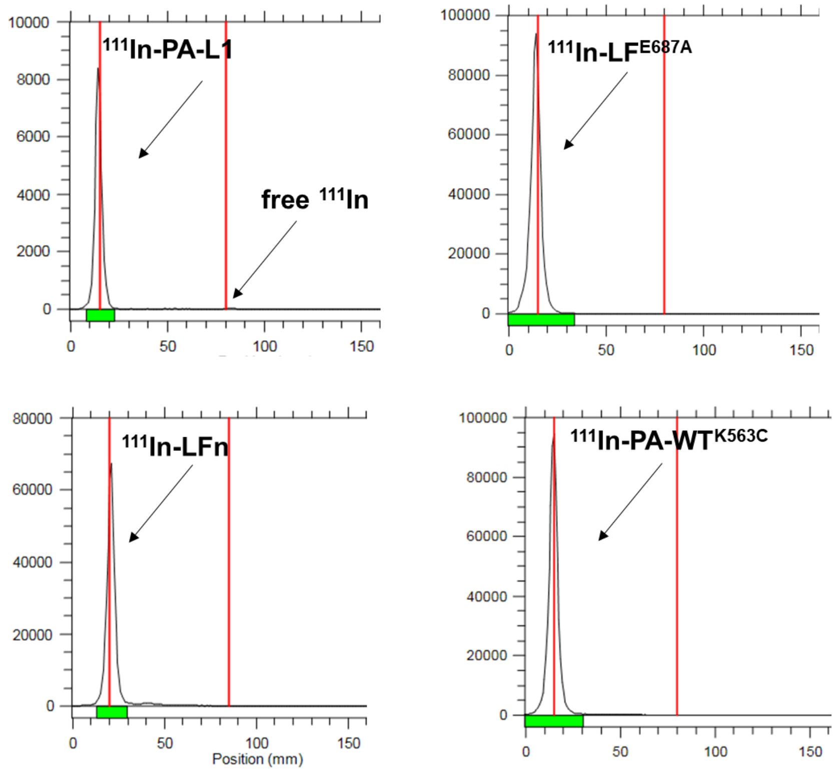

Supplemental Figure 5. Representative radio-iTLC chromatograms for the indicated radiolabelled proteins, showing good radionuclide incorporation and radiochemical purity.

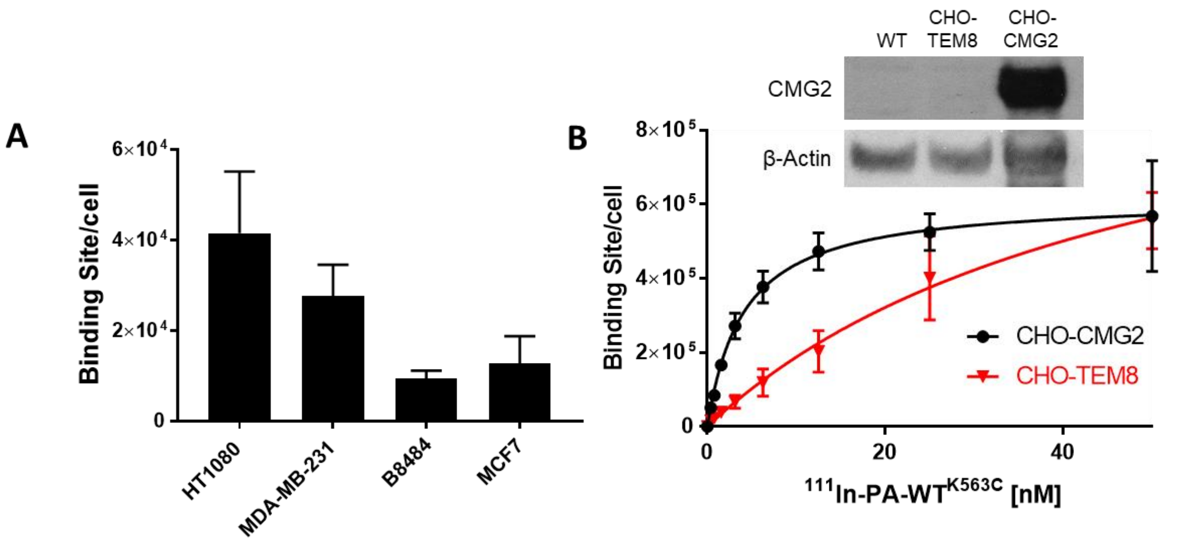

Supplemental Figure 6. Saturation binding assays using ^{111}In-PA-WT^{K563C}. (A) The number of binding sites of PA-WT^{K563C} per cell were calculated from the saturation binding assays. (B) CHO cells transfected to overexpress CMG2 or TEM8 showed binding of ^{111}In-PA-WT^{K563C} to either receptor (K_D = 4.1±0.6 and 50±9.6 nM, respectively). CMG2 expression was evaluated by Western blot.

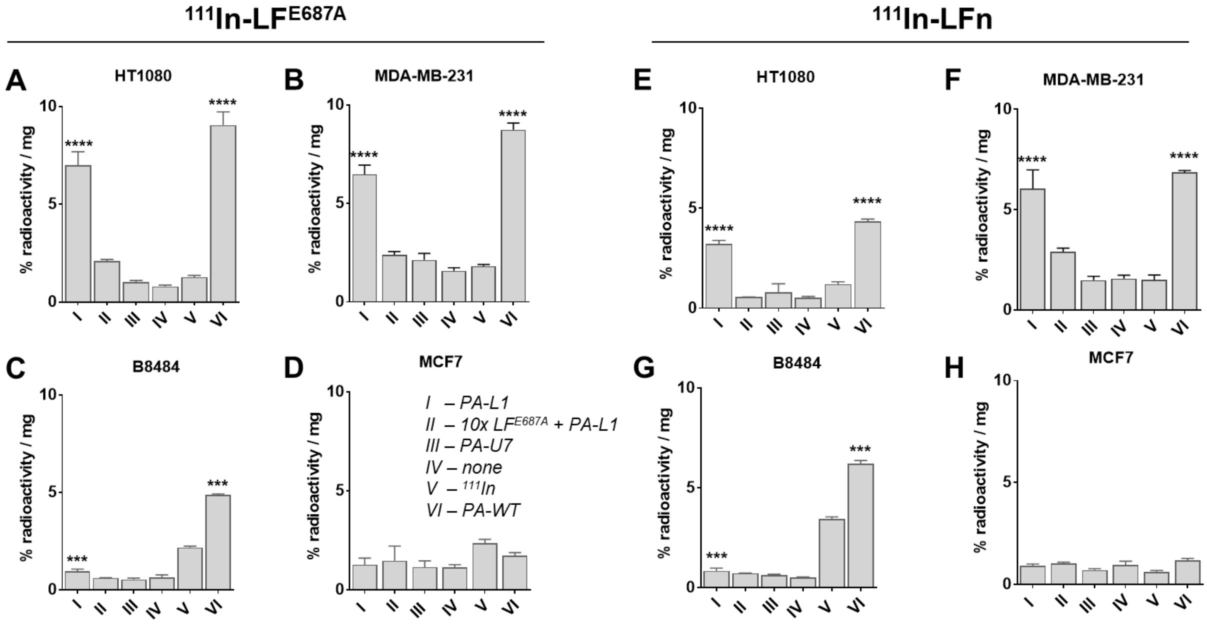

Supplemental Figure 7. Uptake of ^{111}In-LF^{E687A} and ^{111}In-LFn in all cells, demonstrating PA-L1 mediated delivery in all MMP-positive cells, and PA-WT delivery in all furin and CMG2 positive cells. (***P < 0.001; ****P < 0.0001).

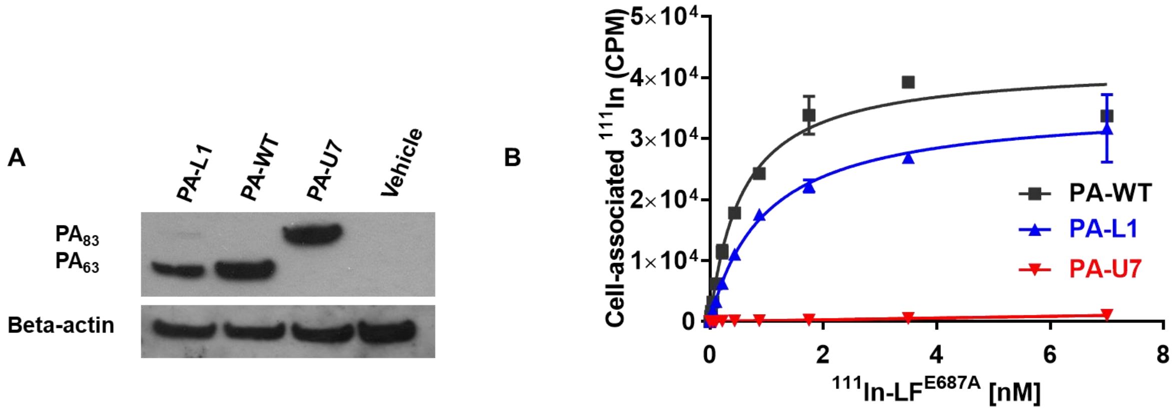

Supplemental Figure 8. ^{111}In-LF^{E687A} binding to PA (pre)pores in CHO-CMG2 cells. CHO-CMG2 cells were exposed to saturating amounts of PAs (PA-WT, PA-L1, or PA-U7) for 24 h at 4°C. (A) Western blot analysis was used to detect PA fragments in CHO-CMG2 lysates, showing PA-L1 and PA-WT are cleaved by CHO-cells, but not PA-U7, which is used as a negative control. (B) Saturation binding assay with increasing amounts of ^{111}In-LF^{E687A} at 4°C for 2 h cells were again washed with cold PBS, solubilized in NaOH, and examined for radioactive content either by gamma counting. Affinity of ^{111}In-LF^{E687E} to PA-L1 pores was slightly less than for PA-WT pores, but not significantly so (P = 0.082).

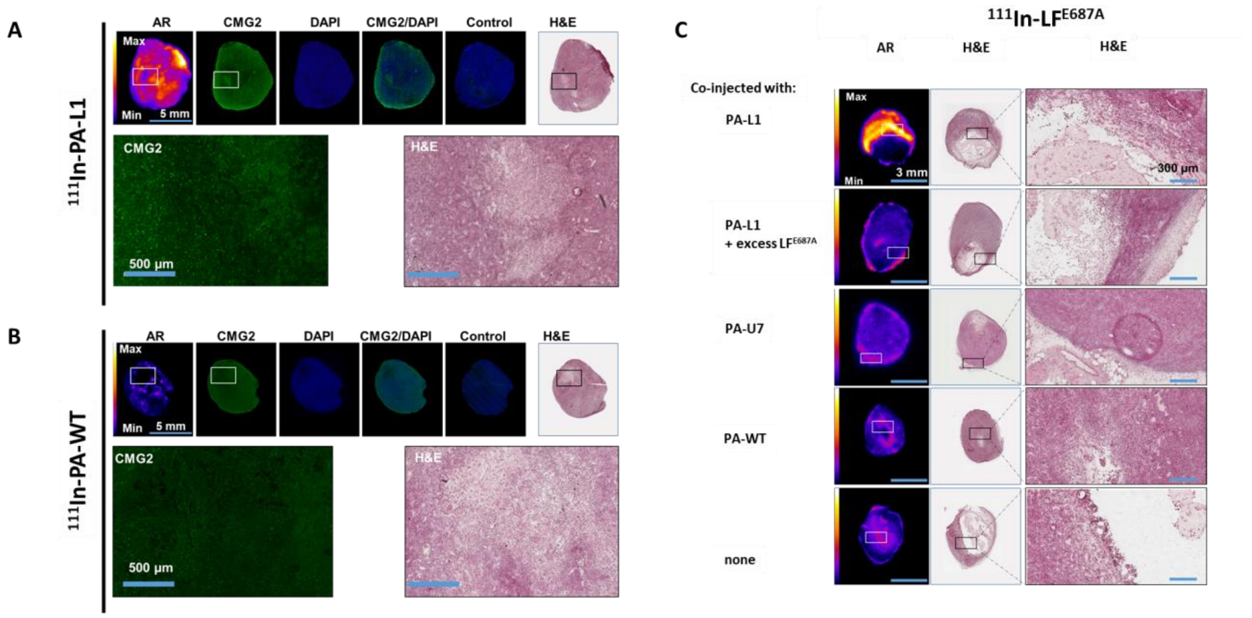

Supplemental Figure 9. Autoradiography of tumor sections obtained from mice bearing MDA-MB-231 tumors injected with (A) ^{111}In-PA-L1, (B) ^{111}In-PA-WT, or (C) ^{111}In-LF^{E687A}, in combination with PAs, as indicated. (scale bar: as indicated)

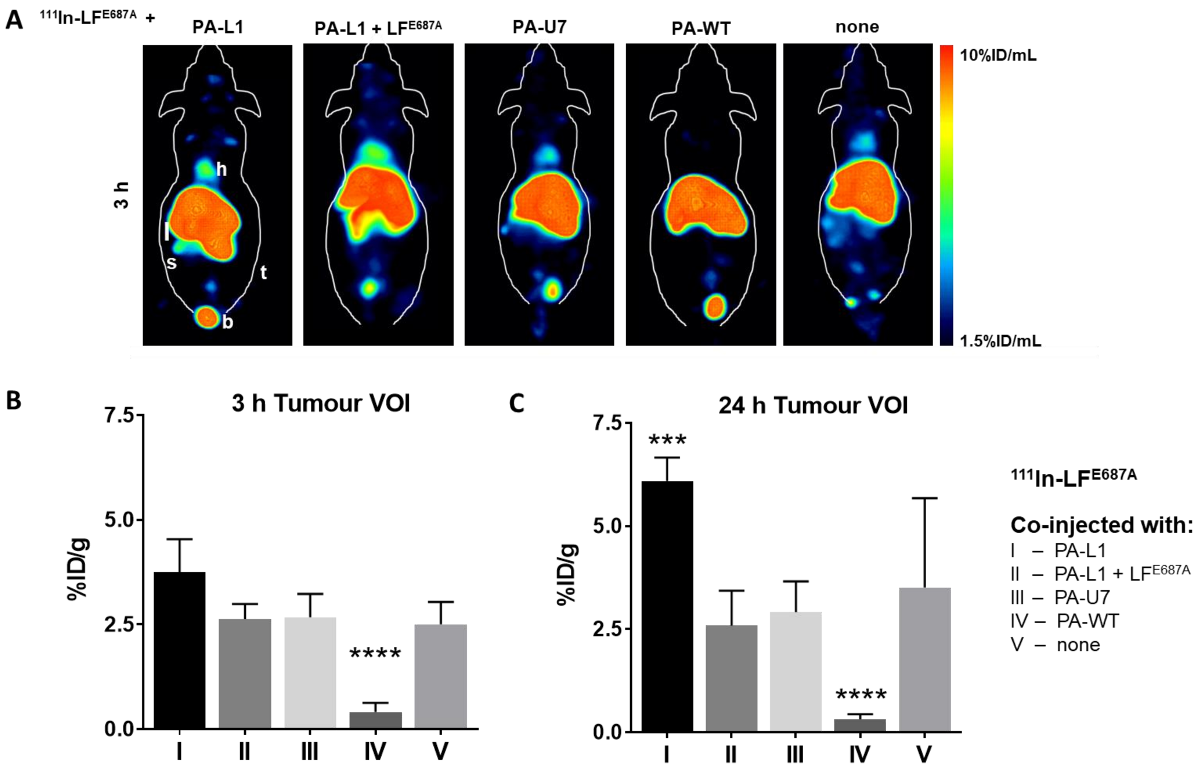

Supplemental Figure 10. (A) Representative SPECT images of MDA-MB-231 tumor-bearing mice, 3 h after intravenous injection with ^{111}In-LF^{E687A}. (B, C) VOI analysis of tumor uptake at 3 and 24 h post injection (h=heart, s=spleen, t=tumor, b=bladder).

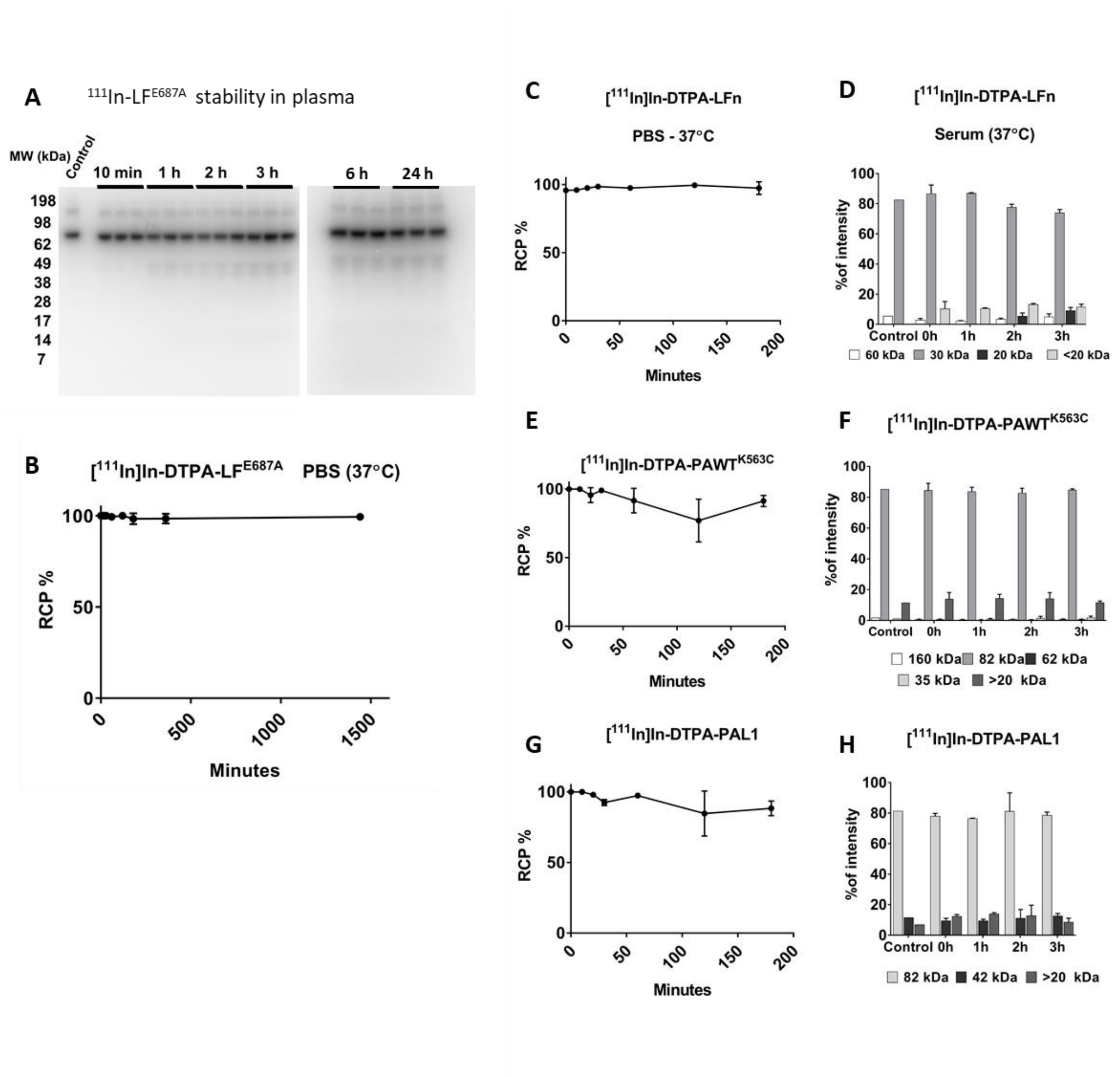

Supplemental Figure 11: Good stability was obtained for ^{111}In-labelled LT components in mouse serum as measured by autoradiography following SDS page (A, D, F, H) or PBS, as measured by ITLC (B, C, E, G).

Supplemental Table 1: Ex vivo biodistribution in naïve animals 1 h and 3 h following intravenous administration of ^{111}In-LFn or ^{111}In-LF^{E687A}.

	**^{111}In-LFn**	**^{111}In-LF^{E687A}**
Blood	0.89 ± 0.08	21.08 ± 3.65
Heart	1.13 ± 0.43	9.22 ± 2.84
Lung	0.97 ± 0.21	8.27 ± 3.02
Liver	5.76 ± 1.04	6.27 ± 2.38
Spleen	3.20 ± 0.24	10.17 ± 4.05
Stomach	0.62 ± 0.42	0.77 ± 0.16
Large intestine	0.49 ± 0.03	0.97 ± 0.31
Small intestine	1.04 ± 0.03	2.53 ± 0.55
Pancreas	0.77 ± 0.07	1.9 ± 0.48
Kidney	28.93 ± 1.03	8.09 ± 1.90
Muscle	0.44 ± 0.38	0.46 ± 0.20
Skin	0.20 ± 0.3	1.35 ± 0.67
Fat	0.15 ± 0.03	1.83 ± 2.24

Supplemental Table 2: Ex vivo biodistribution in mice bearing MDA-MB-231 tumor xenografts at 3 h post administration of ^{111}In-PA-L1 or ^{111}In-PA-WT^{K563C}.

	**^{111}In-PA-L1**	**^{111}In-PA-WT^{K563C}**
Blood	22.23 ±	2.33 ± 1.18
Tumor	5.67 ± 1.69	1.71 ± 0.52
Heart	17.42 ± 1.60	4.91 ± 1.91
Lung	29.84 ± 2.22	11.40 ± 4.45
Liver	22.37 ± 1.04	21.52 ± 2.56
Spleen	30.82 ± 4.80	29.97 ± 12.12
Stomach	2.15 ± 0.83	1.51 ± 1.54
Large intestine	1.92 ± 0.38	0.82 ± 0.23
Small intestine	12.01 ± 1.19	5.23 ± 0.56
Pancreas	3.05 ± 1.55	1.22 ± 0.52
Kidney	184.50 ± 23.58	119.68 ± 27.25
Skin	1.14 ± 0.56	0.58 ± 0.20
Muscle	0.63 ± 0.15	0.21 ± 0.08
Fat	0.69 ± 0.38	0.49 ± 0.44

Supplemental Table 3: Ex vivo biodistribution in mice bearing MDA-MB-231 tumor xenografts at 24 h post administration of ^{111}In-LF^{E687A} in combination with different PAs.

**Group**	I	II	III	IV	V
**^{111}In-LF^{E687A}** +	PA-L1	PA-L1 + LF^{E687A}	PA-U7	PA-WT	none
Blood	0.36 ± 0.05	1.55 ± 0.02	1.26 ± 0.24	0.55 ± 0.05	1.70 ± 0.38
Tumor	5.98 ± 0.62	2.68 ± 0.28	2.74 ± 0.24	1.05 ± 0.21	3.30 ± 1.12
Heart	1.79 ± 0.13	2.98 ± 0.19	2.05 ± 0.39	0.90 ± 0.04	2.48 ± 0.78
Lung	2.26 ± 0.24	3.23 ± 0.27	2.29 ± 0.23	6.18 ± 1.27	2.62 ± 0.75
Liver	53.80 ± 2.08	24.94 ± 1.17	48.03 ± 7.58	62.52 ± 10.08	46.98 ± 2.25
Spleen	51.82 ± 20.34	48.17 ± 2.84	30.92 ± 4.67	126.25 ± 42.81	29.84 ± 16.10
Stomach	0.72 ± 0.20	0.76 ± 0.19	0.82 ± 0.36	0.68 ± 0.13	0.97 ± 0.21
Large intestine	2.60 ± 0.40	3.21 ± 0.55	2.50 ± 0.22	2.77 ± 0.48	3.67 ± 0.36
Small intestine	2.81 ± 0.13	2.03 ± 0.16	1.70 ± 0.25	1.21 ± 0.06	1.87 ± 0.39
Pancreas	0.71 ± 0.18	1.16 ± 0.20	0.96 ± 0.25	0.27 ± 0.11	1.15 ± 0.32
Kidney	5.84 ± 1.79	25.64 ± 3.99	8.60 ± 3.57	5.25 ± 0.26	10.75 ± 2.86
Skin	2.05 ± 1.89	2.16 ± 0.62	1.56 ± 0.09	0.30 ± 0.02	1.35 ± 0.23
Muscle	0.41 ± 0.06	0.54 ± 0.16	0.55 ± 0.05	0.12 ± 0.03	0.50 ± 0.17
Fat	0.79 ± 0.39	1.63 ± 1.11	1.74 ± 0.65	0.50 ± 0.65	1.09 ± 0.36

Supplemental Table 4: Ex vivo biodistribution in mice bearing MDA-MB-231 tumour xenografts at 24 h post administration of ^{111}In-LFn in combination with or without PA-L1.

**Group**	I	V
**^{111}In-LF^{n}** +	PA-L1	none
Blood	0.01 ± 0.01	0.03 ± 0.001
Tumour	0.08 ± 0.01	0.06 ± 0.06
Heart	0.08 ± 0.03	0.09 ± 0.014
Lung	0.06 ± 0.02	0.08 ± 0.02
Liver	1.75 ± 0.10	1.99 ± 0.35
Spleen	0.16 ± 0.18	0.36 ± 0.01
Stomach	0.15 ± 0.08	0.13 ± 0.06
Large intestine	0.36 ± 0.08	0.52 ± 0.05
Small intestine	0.42 ± 0.25	0.24 ± 0.05
Pancreas	0.13 ± 0.001	0.14 ± 0.01
Kidney	14.45 ± 2.30	15.91 ± 0.99
Skin	0.22 ± 1.26	0.04 ± 0.01
Muscle	0.04 ± 0.02	0.05 ± 0.02
Fat	0.10 ± 0.01	0.11 ± 0.09
